# B cell-reactive triad of B cells, follicular helper and regulatory T cells at homeostasis

**DOI:** 10.1038/s41422-024-00929-0

**Published:** 2024-02-07

**Authors:** Yihan Lin, Zurong Wan, Bo Liu, Jiacheng Yao, Tianqi Li, Fang Yang, Jianhua Sui, Yongshan Zhao, Wanli Liu, Xuyu Zhou, Jianbin Wang, Hai Qi

**Affiliations:** 1 Tsinghua-Peking Center for Life Sciences, Beijing, China; 2 Laboratory of Dynamic Immunobiology, Institute for Immunology, Tsinghua University, Beijing, China; 3 Department of Basic Medical Sciences, School of Medicine, Tsinghua University, Beijing, China; 4 Changping Laboratory, Beijing, China; 5 School of Life Sciences, Tsinghua University, Beijing, China; 6 National Institute of Biological Sciences, Beijing, China; 7 Institute of Microbiology, Chinese Academy of Sciences, Beijing, China; 8 New Cornerstone Science Laboratory, School of Medicine, Tsinghua University, Beijing, China; 9 Beijing Key Laboratory for Immunological Research on Chronic Diseases, Tsinghua University, Beijing, China; 10 Beijing Frontier Research Center for Biological Structure, Tsinghua University, Beijing, China; 11 SXMU-Tsinghua Collaborative Innovation Center for Frontier Medicine, Shanxi Medical University, Taiyuan, Shanxi China; 12 Present Address: Weill Cornell Medical College, Cornell University, Ithaca, NY USA

**Keywords:** Autoimmunity, Cell biology

## Abstract

Autoreactive B cells are silenced through receptor editing, clonal deletion and anergy induction. Additional autoreactive B cells are ignorant because of physical segregation from their cognate autoantigen. Unexpectedly, we find that follicular B cell-derived autoantigen, including cell surface molecules such as FcγRIIB, is a class of homeostatic autoantigen that can induce spontaneous germinal centers (GCs) and B cell-reactive autoantibodies in non-autoimmune animals with intact T and B cell repertoires. These B cell-reactive B cells form GCs in a manner dependent on spontaneous follicular helper T (T_FH_) cells, which preferentially recognize B cell-derived autoantigen, and in a manner constrained by spontaneous follicular regulatory T (T_FR_) cells, which also carry specificities for B cell-derived autoantigen. B cell-reactive GC cells are continuously generated and, following immunization or infection, become intermixed with foreign antigen-induced GCs. Production of plasma cells and antibodies derived from B cell-reactive GC cells are markedly enhanced by viral infection, potentially increasing the chance for autoimmunity. Consequently, immune homeostasis in healthy animals not only involves classical tolerance of silencing and ignoring autoreactive B cells but also entails a reactive equilibrium attained by a spontaneous B cell-reactive triad of B cells, T_FH_ cells and T_FR_ cells.

## Introduction

Diverse lymphocyte antigen are generated through somatic recombination, including those that can react to self antigens. More than 50% of newly generated B cells in the bone marrow are estimated to be self-reactive.^1^ Such self reactivities are actively removed from the circulating repertoire by receptor editing, clonal deletion and anergy,^2–5^ three mechanisms that maintain B cell tolerance at the organismal level. Clonal deletion of self-reactive B cells takes place at the immature stage in the bone marrow and at the newly emigrant transitional stage in the spleen, in part because these immature B cells are prone to die in response to receptor crosslinking.^6^ Some mature B cells can be found to react to cytosolic self antigen or possess polyreactivities,^7^ presumably because receptor avidities of such cells for self antigens are too low to trigger cell death or anergy. Tissue-specific self antigens restricted to non-lymphoid organs are physically segregated from autoreactive B cells and do not cause deletion or anergy. These autoreactive B cells are ignorant B cells.^8,9^

From a perspective of cellular dynamics, one can envision that, for some newly emigrant autoreactive B cells, self antigen may only become abundantly available in a sufficiently stimulatory form inside the B cell follicle. In this hypothetical scenario, by reaching the follicle, autoreactive B cells have passed clonal deletion and anergy checkpoints and are fully competent. No known B cell-intrinsic tolerance mechanism could prevent such cells from mounting an active response to follicular self antigen. Furthermore, mature follicular B cells spend a significant amount of their lifetime in the follicle.^10^ The dense packing of B cells in the follicle, as opposed to elsewhere in the body that B cells might traverse, creates an environ in which B cell-derived autoantigens (BDAs) may constitute a type of follicular self antigen postulated above. BDAs may be so concentrated in the follicle that, while autoreactive B cells capable of recognizing BDAs are not triggered for deletion or anergy induction outside of the follicle, they become activated inside the follicle. Molecules expressed on the B cell surface are of particularly concerns, because as autoantigen they do not require cell damage or cell death to be released and are naturally displayed on a 2-dimentional surface essentially spanning the entire follicle (Supplementary information, Fig. S1), a condition very much conducive to B cell activation. In theory, after antigen receptor triggering, BDA-recognizing B cells would follow the same itinerary as foreign antigen-activated follicular B cells to relocate to the T zone-follicle border.^11,12^ They could die there if cognate T cell help is limited.^13^ However, clonal deletion of T cells recognizing tissue-restricted autoantigen is far from complete.^14,15^ As a result, follicular activation of BDA-recognizing B cells may lead to a bona fide T-dependent autoreactive B cell response in normal animals. In the current study, we explored this hypothesis and showed that BDA-specific GC responses under regulatory control characterized the immune homeostasis.

## Results

A T-dependent B cell response involves germinal centers (GCs), in which antigen-activated B cells proliferate, hypermutate, and undergo affinity-based selection by follicular T helper (T_FH_) cells.^16,17^ While the GC reaction is a hallmark of active immune responses to foreign antigens following vaccination or infections, GCs also spontaneously form in mice genetically predisposed to autoimmunity^18^ and even in normal mice.^19^ In the latter case, microbiota, food components and other environmental materials are plausible source of antigen, while bona fide autoantigen cannot be ruled out. Therefore, we decided to look for BDA-reactive B cells and T cells in GCs that spontaneously develop in healthy, unmanipulated animals.

### Spontaneous GC development

**Fig. 1:**
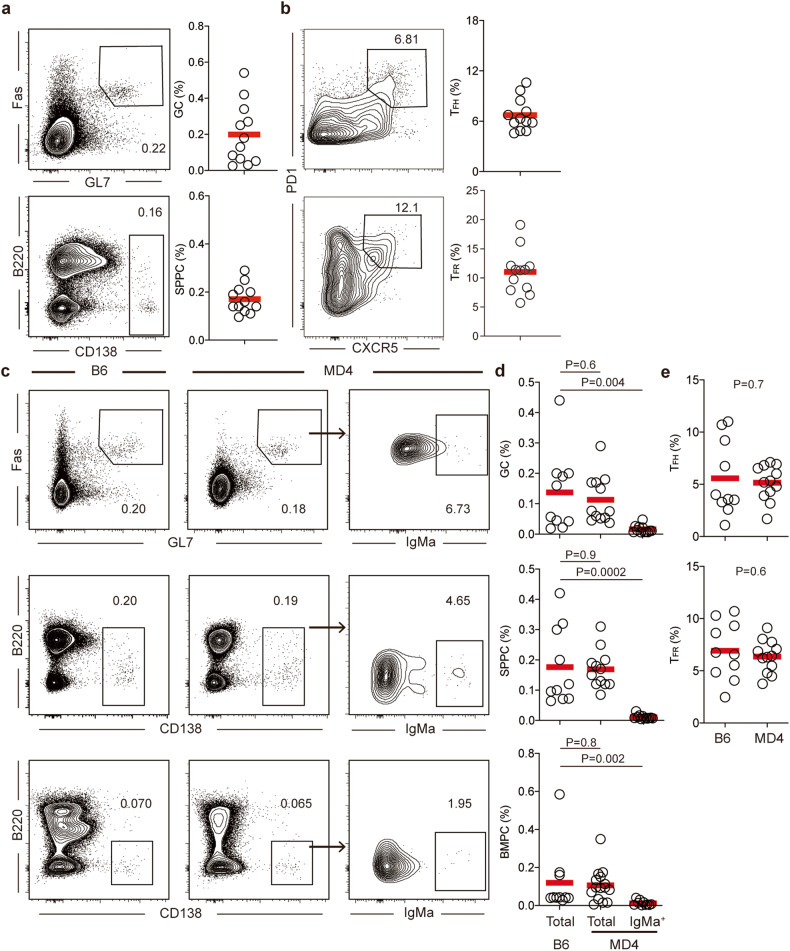
Spontaneous splenic GC responses. **a**, **b** Spontaneous GCs in WT B6 mice of 2–3 months of age. **a** Representative contour plots and summary statistics of GC and SPPC frequencies in B220^+^ B cells and total splenocytes, respectively. **b** Spontaneous T_FH_ cells in CD4^+^CD44^hi^Foxp3^*−*^ and T_FR_ cells in CD4^+^CD44^hi^Foxp3^+^ cells. **c**–**e** Spontaneous GCs in B6 and MD4 mice of 2–3 months of age. **c** Representative contour plots and summary statistics of GC, SPPC and BMPC frequencies in B220^+^ B cells, total splenocytes and total BM cells, respectively. For MD4 mice, IgMa^+^ cells in GC or plasma cells as identified by surface or intracellular staining are also shown. **d** Summary statistics of total GCs, SPPCs and BMPCs in B6 and MD4 mice and IgMa^+^ GCs, SPPCs and BMPCs in MD4 mice. **e** Summary statistics of T_FH_ and T_FR_ frequencies in CD4^+^CD44^hi^Foxp3^*−*^ and CD4^+^CD44^hi^Foxp3^+^ cells. Each symbol represents one mouse, and lines denote mean values. Data were pooled from three independent experiments. *P* values by Mann–Whitney tests.

Extending previous findings,^19^ we found that, in specific pathogen-free (SPF) B6 mice of 8–10 weeks of age, ~0.2% of total B220^+^ B cells were Fas^+^GL7^+^ GC B cells, and ~0.2% of total splenocytes were plasma cells (SPPC; Fig. 1a). These frequencies of splenic GCs and SPPCs are consistent with what are generally reported in literature as the background or control condition of unmanipulated animals. At these frequencies, GCs could be seen histologically as sporadic clusters of Ephrin B1-expressing cells^20,21^ in some but not all B-cell follicles (Supplementary information, Fig. S2). These spontaneous GCs were accompanied by appearance of CD4^+^CD44^+^CXCR5^hi^PD-1^hi^FoxP3^*−*^ T_FH_ and CD4^+^CD44^+^CXCR5^hi^PD-1^hi^FoxP3^+^ T_FR_ cells (Fig. 1b). We further examined MD4 mice that carry transgenic IgMa B cell receptor (BCR) recognizing hen egg lysozyme. Spontaneous GCs, SPPCs, and bone marrow PCs (BMPC) frequencies were actually comparable between co-housed MD4 and B6 littermates (Fig. 1c, d), and so were frequencies of spontaneous T_FH_ and T_FR_ cells (Fig. 1e). Importantly, however, because essentially none of spontaneous GC, SPPC, and BMPCs in MD4-transgenic animals expressed IgMa MD4 BCR or antibodies (Fig. 1d), these cells must carry BCRs generated by V(D)J recombination at the endogenous immunoglobulin (Ig) locus. Considering the stringent allelic exclusion exerted by transgenic IgMa MD4 BCR, these results support the possibility that spontaneous GCs in a non-autoimmune background may include autoantigen-driven components.

### Spontaneous GCs depend on T cell help and are constrained by T_FR_ cells

To determine whether spontaneous GCs depend on T-cell help just like conventional GCs induced following immunization or infection, we examined wild-type (WT) B6 mice and mice deficient in SLAM-associated protein (SAP) that is essential for cognate T–B interactions and GC formation.^22,23^ As shown in Supplementary information, Fig. S3a, 3- to 4-month-old WT mice had ~0.5% of total B cells as GCs, while SAP-deficient mice had essentially none. SPPCs were also significantly reduced in the absence of SAP (Supplementary information, Fig. S3b). Moreover, CD40L blockade essentially abrogated spontaneous GCs in 4 days, while SPPCs was also slightly reduced as a consequence in the same time frame (Supplementary information, Fig. S3c, d). Therefore, spontaneous GCs depend on conventional T-cell help.

Regulatory T (Treg) cells enforce dominant tolerance extrathymically, in part by suppressing activation of self-reactive T cells.^24–26^ Follicular T regulatory (T_FR_) cells are the Treg subset that regulates the GC response.^27–29^ Similar to T_FH_ cells, T_FR_ cells exhibit a CXCR5^+^PD-1^+^ surface phenotype, are localized in the B-cell follicle, and express both the Treg-defining Foxp3 and the T_FH_-defining Bcl6 transcription factor.^30–32^

**Fig. 2:**
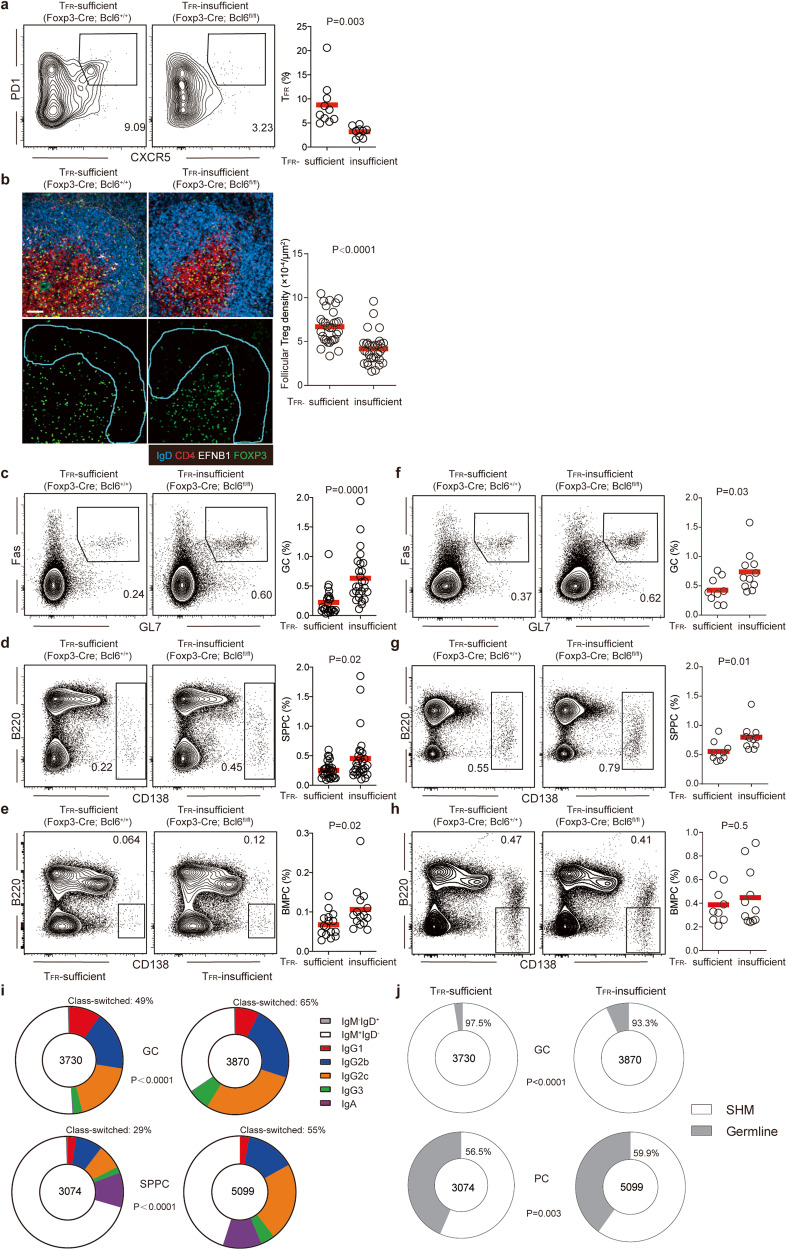
Spontaneous GCs are constrained by T_FR_ cells. **a**, **b** Validation of T_FR_ insufficiency. **a** Representative contour plots and summary statistics of CD4^+^CD44^hi^CXCR5^+^PD-1^+^Foxp3^+^ T_FR_ cells in CD4^+^CD44^hi^Foxp3^+^ cells in 2- to 3-month-old mice of indicated genotypes. **b** Representative images (left) and summary statistics (right) of follicular T_FR_ distribution in mice of indicated genotypes. Scale bar, 50 μm. Each symbol represents one mouse (**a**) or follicle (**b**), and lines denote mean values. Data were pooled from three independent experiments. *P* values by two-tailed unpaired *t*-tests. **c**–**h** Representative contour plots and summary statistics of GC frequencies in B220^+^ B cells (**c**, **f**), SPPCs in total splenocytes (**d**, **g**) and BMPCs in total bone marrow cells (**e**, **h**) in mice of 2 to 3 months (**c**–**e**) or 10 to 12 months (**f**–**h**) of age. Each symbol represents one mouse, and lines denote mean values. Data were pooled from two (**f**–**h**) or three (**e**) or five (**c**, **d**) independent experiments. *P* values by two-tailed unpaired *t*-tests. **i**, **j** Isotype distribution (**i**) and somatic hypermutation (SHM) fraction (**j**) of GCs and SPPCs by single-cell BCR sequencing. Shown are proportions of indicated components in GCs (two pie charts on the top) and SPPCs (two on the bottom) in mice of indicated genotypes. Total numbers of cells sequenced are shown at the center. Cells were pooled from 8 T_FR_-sufficient and 10 T_FR_-insufficient mice of 2–3 months of age, respectively. *P* values by Fisher’s exact tests.

To determine whether spontaneous GCs are constrained by T_FR_ cells, we used Treg reporter FOXP3-GFP-hCre (called “Foxp3-cre”) mice.^33^ As shown in Fig. 2a, under the SPF condition, ~10% CD44^hi^GFP^+^ Treg cells in these mice co-expressed CXCR5 and PD-1, exhibiting a T_FR_ phenotype. In Foxp3-cre;*Bcl6*^*fl/fl*^ mice, in which the *Bcl6* gene is specifically ablated in FoxP3-expressing Treg cells, the FoxP3^+^CXCR5^hi^PD-1^hi^T_FR_ frequency was markedly reduced. By immunohistochemical staining, B-cell follicles in Foxp3-cre mice contained GFP^+^ T_FR_ cells, whereas much fewer were seen in follicles of Foxp3-cre;*Bcl6*^*fl/fl*^ mice (Fig. 2b). For simplicity, we hereafter refer to Foxp3-cre;*Bcl6*^*fl/fl*^ mice as T_FR_-insufficient mice. At 2–3 months of age, T_FR_-insufficient mice showed a 2- to 3-fold increase in spontaneous GCs, SPPCs, and BMPCs, as compared to their WT counterparts (Fig. 2c–e). As mice aged to 10–12 months, WT mice harbored twice to three times as many spontaneous GCs and SPPCs and four times as many BMPCs as their 2- to 3-month-old counterparts, while T_FR_-insufficient mice continued to show a two-fold increase over the WT in terms of GCs and SPPCs but not BMPCs, which amounted to ~0.4% of total BM cells in both groups of mice (Fig. 2f–h). This latter observation is consistent with the notion of BMPC survival niche of a finite size.^34^ In addition, receptor sequencing by 10× Genomics showed that, in T_FR_-insufficient mice, a significantly higher fraction of spontaneous GCs was isotype-switched, and this increase was particularly pronounced in the SPPC compartment (Fig. 2i). With or without intact T_FR_ cells, most of spontaneous GC B cells harbor somatic mutations (Fig. 2j).

Therefore, similar to conventional GCs induced by immunization or infection, spontaneously formed GCs are dependent on T cell help, constrained by T_FR_ cells, and involve somatic hypermutation and class-switching.

### Spontaneous GCs harbor specificities against B cell-derived autoantigen

**Fig. 3:**
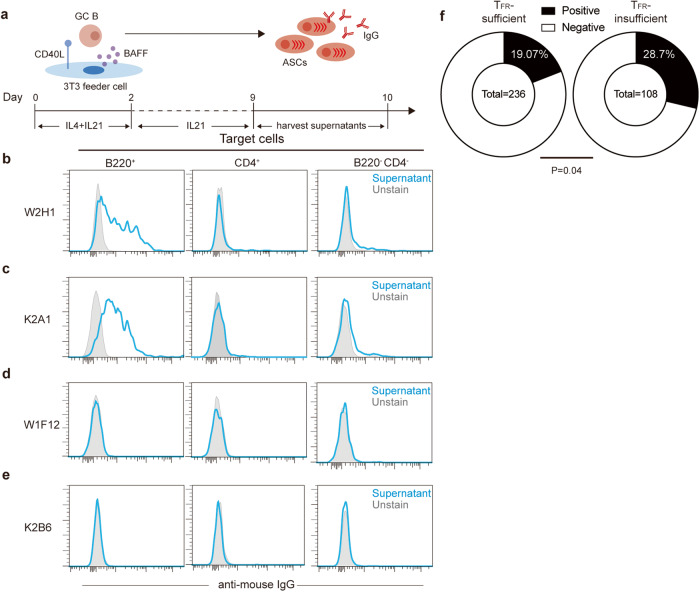
Spontaneous GCs recognize B cell-surface autoantigen. **a** The protocol for cloning single spontaneous GC B cells from 2–3-month-old T_FR_-sufficient and -insufficient mice to produce antibody-secreting cells (ASCs) in vitro. **b**–**e** Histogram profiles of representative ASC supernatants that were derived from TFR-sufficient (**b**, **d**) or -insufficient (**c**, **e**) GCs and did (**b**, **c**) or did not (**d**, **e**) stain the B cell surface, measured with splenocytes from MD4 mice as target cells. **f** Frequencies of BDA-reactive ASC supernatants. Total numbers of clones analyzed are shown at the centers of pie charts. *P* value by the χ^2^ test.

To explore whether spontaneous GCs contain specificities against BDAs, we set up Nojima culture^35,36^ to clone and convert single GC B cells to antibody-producing cells (Fig. 3a). The culture supernatant was taken at day 9 and 10 from each well and used to test reactivities to surface molecules of B cells by flow cytometry. MD4 B cells were used as the test target in an effort to minimize potential interference by polyclonal BCR idiotypes.

We cloned 236 spontaneous GC B cells from the WT and 108 from T_FR_-insufficient mice. After IgG concentrations in those culture supernatants were normalized to 2 μg/mL, they were directly tested as the primary staining reagent, with an anti-mouse IgG antibody as the secondary. As exemplified in Fig. 3b, c, many supernatants of GC clones stained components on the B cell surface but showed far less or not at all staining on CD4^+^ or B220^−^CD4^−^ splenocytes, while other clones did not react to B cells (Fig. 3d, e). Such B cell-reactive clones accounted for ~19% and ~29% in spontaneous GCs of WT and T_FR_-insufficient mice, respectively (Fig. 3f; see Supplementary information, Fig. S4 for staining histograms of all tested antibody supernatants). These data indicate that at least 20% of spontaneous GCs recognize B cell-derived surface components and that T_FR_ cells restrain these cells.

### FcγRIIB is an autoantigen for spontaneous GCs

**Fig. 4:**
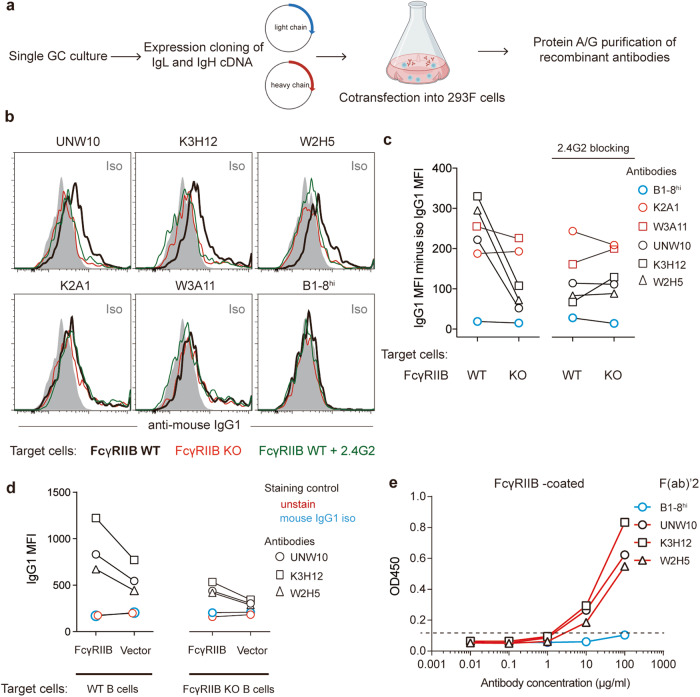
FcγRIIB is an autoantigen for spontaneous GCs. **a** The protocol for producing recombinant antibodies. **b** Histogram profiles of staining B220^+^IgD^+^ B cells of indicated genotypes with 3 spontaneous GC-derived and B1-8hi BCR-derived recombinant antibodies, with or without 2.4G2 blockade. **c** Mean fluorescence intensities (MFI) of staining as in **a**. **d** MFIs of staining B cells that were transduced with FcγRIIB or vector control with the 3 spontaneous GC-derived recombinant antibodies or mouse IgG1 isotype control. **e** Direct ELISA showing binding of indicated recombinant F(ab)’_2_ antibodies to the FcγRIIB extracellular domain. One of two independent experiments with similar results are shown.

In an effort to positively identify surface BDAs, we cloned Ig heavy and light chains from 13 single GC B cells that produced B cell surface-binding antibodies (Fig. 4a; Supplementary information, Table S1). When these IgH/L pairs were expressed in 293 F cells as recombinant IgG1 antibodies, they all demonstrated an ability to recognize antigen on the B cell surface. Interestingly, we found that 3 antibodies (UNW10, K3H12 and W2H5) isolated from different mice were reactive to FcγRIIB, the low-affinity IgG Fc receptor that binds to IgG immune complexes and transmit inhibitory signals to B cells.^37^ As exemplified in Fig. 4b and quantified in Fig. 4c, those 3 antibodies (20 μg/mL) can positively stain WT but not FcγRIIB-deficient B220^+^IgD^+^ naïve B cells, and the staining was abrogated by pre-incubation of the cells with the 2.4G2 anti-FcγRII/III antibody. On the other hand, K2A1 and W3A11, two additional surface BDA-reactive antibodies expressed in the same IgG1 format and tested at the same 20 μg/mL concentration, positively stained both WT and FcγRIIB-deficient B220^+^IgD^+^ naïve B cells to a similar extent, and the staining was not affected by 2.4G2 pre-treatment. Recombinant 4-hydroxy-3-nitrophenyl (NP) hapten-specific B1-8hi antibody of the same IgG1 isotype did not show any staining of the B cell surface above the background level achieved with non-specific mouse IgG1 (Fig. 4b, c). Moreover, when mouse FcγRIIB was retrovirally expressed in WT or FcγRIIB-deficient B cells (Supplementary information, Fig. S5), staining of such cells with UNW10, K3H12 and W2H5 was markedly higher than staining of vector-transduced cells (Fig. 4d). These data indicate that these 3 antibodies uniquely recognize FcγRIIB as antigen.

To completely rule out the remote possibility that the observed differential binding was because, by unknown mechanisms, the FcγRIIB receptor could preferentially recognize Fc portions of UNW10, K3H12 and W2H53 antibodies but not K2A1, W3A11 or B1-8hi antibodies, we made recombinant F(ab)’_2_ versions of UNW10, K3H12 and W2H5 and control B1-8hi antibodies. By direct ELISA, UNW10, K3H12 and W2H5 but not B1-8hi F(ab)’_2_ were found to bind to the extracellular domain of the FcγRIIB receptor (Fig. 4e).

FcγRIIB is constitutively expressed by naïve follicular B cells and is upregulated on follicular dendritic cells after GC formation,^38^ making FcγRIIB an abundant follicular autoantigen. Among UNW10, K3H12 and W2H5 antibodies, except for K3H12 harboring 2 somatic mutations that cause an amino acid replacement in the IgL complementarity-determining region 3 (CDR3), there is no somatic mutation in UNW10 and only a synonymous mutation in W2H5 (Supplementary information, Table S1). We can thus rule out the possibility that the FcγRIIB reactivity was acquired through somatic hypermutation in a GC response against unknown environmental antigen. We conclude that FcγRIIB is a BDA, and B cells specific to this BDA can readily develop into GCs spontaneously in non-autoimmune mice.

### Spontaneous T_FH_ and T_FR_ cells preferentially recognize B cell autoantigen

Given the proof of concept provided by the FcγRIIB as a BDA and the estimated > 20% prevalence of BDA specificities in spontaneous GCs, we suspect spontaneous T_FH_ cells would have to recognize B cell-derived autoantigen so as to provide cognate help. As T_FR_ cells clearly constrain spontaneous GCs, spontaneous T_FR_ cells may also preferentially recognize BDAs.

**Fig. 5:**
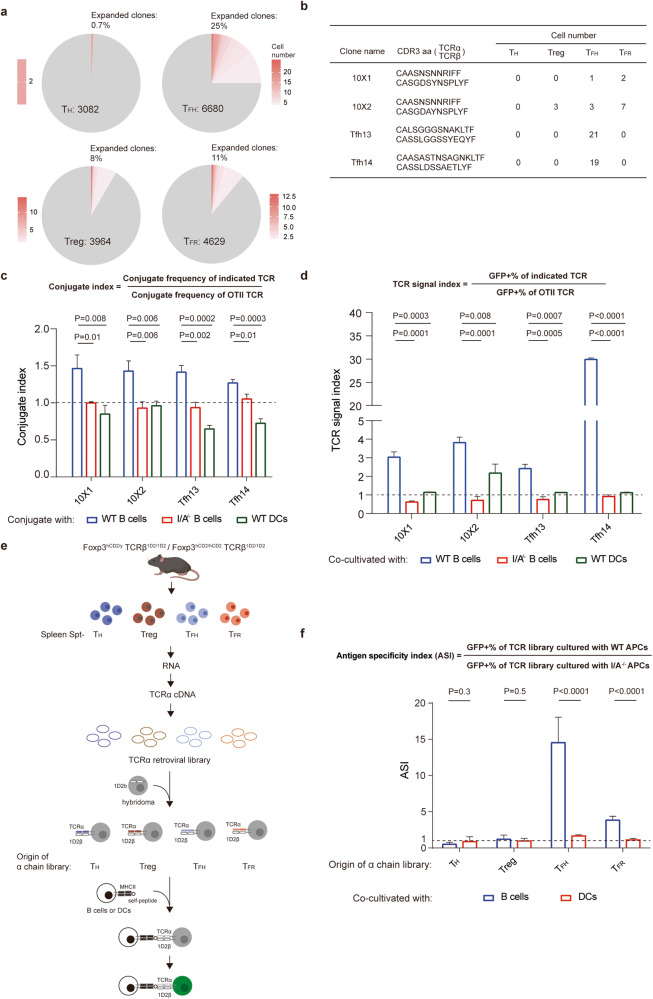
Spontaneous T_FH_ and T_FR_ cells preferentially recognize B-cell autoantigen. **a** Clonal characteristics of T_H_ (FoxP3-GFP^–^CD4^+^CD44^+^CXCR5^–^PD-1^–^), T_FH_ (FoxP3-GFP^–^CD4^+^CD44^+^CXCR5^+^PD-1^+^), Treg (FoxP3-GFP^+^CD4^+^CD44^+^CXCR5^–^PD-1^–^), and T_FR_ (FoxP3-GFP^+^CD4^+^CD44^+^CXCR5^+^PD-1^+^) cells. The gray color indicates unique TCR clones without observed repeats, while the red color of increasing shades indicates increasing clonal size (in cell number) of the same TCR. Total numbers of cells sequenced are given in the pie chart. Data were pooled from 5 independent experiments or meta-mice sequenced separately, each of which contained a pool of 8 Foxp3-IRES-GFP mice of 2–3 months of age. **b** Four TCRs (10 × 1, 10 × 2, Tfh13 and Tfh14) analyzed in detail below, with their CDR3 sequences and their observed counts in indicated cell populations. **c** Conjugate indices of the four TCRs, with WT B cells (blue), class II MHC-deficient B cells (red) or WT DCs (dark green) as the APC partner. Raw data of conjugate frequencies are provided in Supplementary information, Fig. S6. The horizontal dashed line indicates index 1, at which a TCR confers a conjugation efficiency equal to that of the OT-II TCR. One of two independent experiments with similar results are shown. Bars indicates the standard deviation (STD) of triplicated samples. *P* values by two-tailed unpaired *t-*tests. **d** TCR signal indices of the four TCRs, as assayed in NFAT-GFP reporter hybridoma stimulated with WT B cells (blue), class II MHC-deficient B cells (red) or WT DCs (dark green). Raw data of NFAT-GFP^+^ frequencies are provided in Supplementary information, Fig S7. One of two independent experiments with similar results are shown. Bars indicates STD of triplicated co-culture wells. *P* values by two-tailed unpaired *t*-tests. **e** The protocol for constructing TCR libraries and testing antigen specificity preference of receptor repertoires (more details in Materials and methods). **f** Antigen specificity index (ASI) of TCR libraries from indicated T cell populations, as assayed in NFAT-GFP reporter hybridoma stimulated with WT or class II MHC-deficient B cells (blue) or DCs (red). One of two independent experiments with similar results are shown. Bars indicate STD of 4–6 co-culture wells. *P* values by two-tailed unpaired *t*-tests.

To test BDA-directed specificities, we sequenced T cell receptor (TCR) α and TCRβ chains from sorted single cells by 10× Genomics (see Supplementary information, Fig. S6 for the sorting strategy using Foxp3-IRES-GFP mice). As shown in Fig. 5a, in young adult mice of 2–3 months of age, ~25% of spontaneous T_FH_ (FoxP3-GFP^−^CD4^+^CD44^+^CXCR5^+^PD-1^+^) cells were clonally expanded (identical pairs of TCRα and TCRβ DNA sequences; 1667 of 6680 cells), in contrast to ~0.7% in non-T_FH_ (FoxP3-GFP^−^CD4^+^CD44^+^CXCR5^*−*^PD-1^*−*^)-activated T_H_ cells (22 of 3082). On the other hand, T_FR_ (FoxP3-GFP^+^CD4^+^CD44^+^CXCR5^+^PD-1^+^) cells and other Treg (FoxP3-GFP^+^CD4^+^CD44^+^CXCR5^*−*^PD-1^*−*^) cells contained ~11% and ~8% expanded clones, respectively (512 of 4629 T_FR_ cells vs 332 of 3964 Treg cells). These data indicate more frequent clonal expansion of spontaneous T_FH_ and T_FR_ cells as compared to their respective non-follicular counterparts, suggestive of active and ongoing antigen stimulation that these cells experience in the follicle.

As shown in Fig. 5b and Supplementary information, Table S2, we further identified αβ TCRs with identical amino acid sequences that appeared in T_FH_ and T_FR_ cells (e.g., 10 × 1) or in T_FH_, T_FR_ and Treg cells (e.g., 10 × 2). 10 × 1 and 10 × 2 share the same TCRα CDR3 amino acid sequence, and they differ by only one amino acid in TCRβ CDR3 sequences. 10 × 2 TCR was observed in different meta-mice or individual mice that were barcoded and analyzed in multiple separate experiments (see Materials and methods for details). Importantly, 10 × 2 manifested itself in different DNA sequences in different mice or meta-mice (Supplementary information, Table S2) and even between T_FH_ and Treg cells from the same mice (i.e., the 5-4 mouse, Supplementary information, Table S2), indicating a strong selection driven by an abundant common autoantigen. Tfh13 and Tfh14 were the most and the second most abundant clones in spontaneous T_FH_ cells, respectively, and they were not found in T_FR_ or Treg cells (Fig. 5b). Tfh13 was shared by 5-1 mouse and a meta-mouse (meta-3; Supplementary information, Table S2), again indicating selection by common autoantigen.

By retroviral transduction into polyclonal B6 T cells, we tested reactivities of those four TCRs toward B cells. We reason that if these TCRs recognize epitopes derived from B cell autoantigen, such epitopes should be naturally presented by B cells themselves. Therefore, we examined direct conjugate formation between TCR-transduced T cells and WT B cells or B cells deficient in class II major histocompatibility complex (MHC) expression. As compared to control OT-II TCR that was expressed in B6 T cells, 10 × 1, 10 × 2, Tfh13 and Tfh14 TCRs afforded a significant increase in conjugation with WT B cells; such advantage over OT-II TCR was abrogated when class II MHC-deficient B cells were used (Fig. 5c; Supplementary information, S7a, b). When splenic dendritic cells (DCs) were used as the antigen-presenting cells (APCs), those 4 TCRs did not show any advantage over OT-II TCR in conjugation (Fig. 5c; Supplementary information, S7c). These data support that those 4 TCRs recognize BDAs.

To corroborate these findings, we reconstituted mhCD4-NFAT-GFP T cell hybridoma^39^ (see Materials and methods for details) with those four TCRs or control OT-II TCR. While four test TCRs were expressed at comparable or slightly lower levels than the control OT-II TCR (Supplementary information, Fig. S8a), they all gave rise to more GFP^+^ cells than OT-II, 24 h after hybridoma cells were co-cultivated with WT B cells but not with class II MHC-deficient B cells (Fig. 5d; see Supplementary information, Fig. S8b–d for cytometry profiles and raw quantitation). When DCs were used as APCs, only 10 × 2 showed a detectable but weaker response while the other three did not detectably respond (Fig. 5d; Supplementary information, Fig. S8b, e). The fact that these TCR-reconstituted hybridoma cells responded more strongly to B cells was not because B cells were generally better APCs, as OT-II TCR-reconstituted hybridoma cells responded much stronger to DCs than B cells when exogenous OVA_323-339_ peptide was used as the antigen (Supplementary information, Fig. S8f). Therefore, all 4 TCRs isolated from spontaneous T_FH_ or T_FR_ cells preferentially react to B cell-derived but not DC-derived autoantigen.

Encouraged by these results, we systemically tested whether spontaneous T_FH_ and T_FR_ cell populations are selectively enriched with TCR specificities against BDAs. We took advantage of the 1D2 mouse strain that has been created through nuclear transfer of the nucleus from a T cell of unknown specificity^40^ (a gift from Dr. Shohei Hori). As a result, the 1D2 strain has a recombined *Tcrb* locus expressing β chain of the 1D2 TCR. The β chain-fixed 1D2 strain was further bred with the FoxP3-hCD2 knock-in reporter strain to obtain *Tcrb*^*1D2/1D2*^*Foxp3*^*hCD2/y*^ or *Tcrb*^*1D2/1D2*^*Foxp3*^*hCD2/hCD2*^ mice (Fig. 5e). From these mice, we isolated spontaneous T_FH_ cells, T_FR_ cells, conventional non-T_FH_ activated T cells, and Treg cells (Supplementary information, Fig. S9a) to construct their respective α chain libraries. These α chain libraries were then separately transduced into an NFAT-GFP hybridoma subline that was engineered to stably express 1D2 TCR β chain (Fig. 5e). While such 1D2 β chain-modified hybridoma does not express TCR on the cell surface, introduction of a TCRα chain led to receptor complementation and surface expression of TCR (Supplementary information, Fig. S9b). After 24 h culture of these TCR-reconstituted hybridoma cells together with WT or class II MHC-deficient B cells or DCs, GFP^+^ frequencies were enumerated in each group (Supplementary information, Fig. S9c–f). By dividing the frequency of GFP^+^ hybridoma cells after stimulation with WT APCs by the GFP^+^ frequency achieved with MHC-deficient cells, we define the antigen specificity index (ASI), which reflects the extent to which a polyclonal TCR repertoire can react to autoantigen presented by APCs. As shown in Fig. 5f, TCRs from non-T_FH_ activated T cells or non-T_FR_ Treg cells exhibited an ASI close to 1, no matter whether B cells or DCs were the APC. In striking contrast, TCRs from spontaneous T_FH_ cells exhibited an ASI close to 15 in response to B cells, and ASI of TCRs from spontaneous T_FR_ cells was ~4 in response to B cells. In response to autologous DCs, spontaneous T_FH_ but not T_FR_ TCRs showed a marginally detectable antigen-specific response (Supplementary information, Fig. S9e, f; Fig. 5f). Notably, among the four types of T cells analyzed (T_FH_, T_H_, T_FR_, and Treg cells), the ASI or the strength of their TCR repertoires reacting to B cells matched the relative extent of clonal expansion these cells exhibited (Fig. 5a). Taken together, these data indicate that spontaneous T_FH_ cells and T_FR_ cells are selectively enriched with TCR specificities for BDAs.

In summary, in normal mice with an unmanipulated receptor repertoire, the immune system does not silence all autoreactive clones, particularly not BDA-reactive ones. Instead, follicular homeostasis is maintained in association with ongoing T-dependent GC responses to BDAs.

### BDA-specific GCs persist and expand following infection

**Fig. 6:**
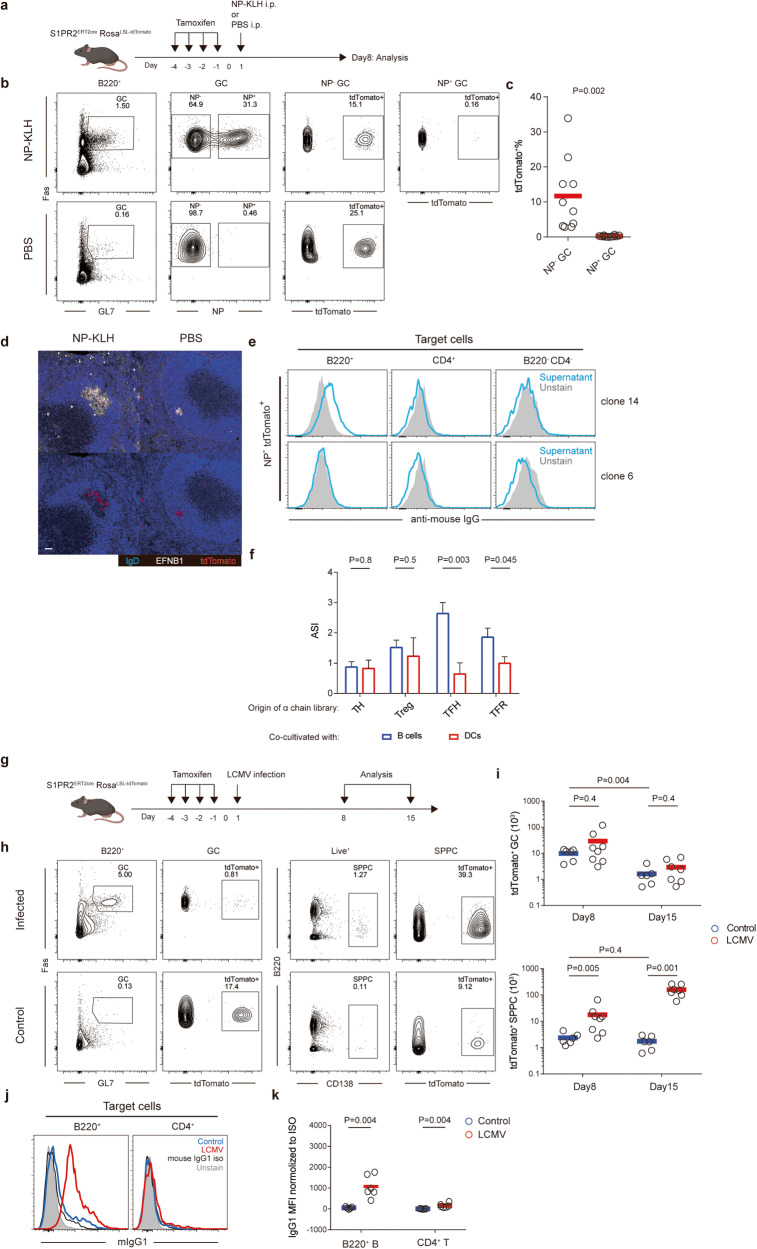
GC merging and increase in BDA-specific antibodies during virus infection. **a** The protocol for labeling spontaneous GCs prior to immunization. **b** Representative flow-cytometry profiles, showing GCs in B220^+^ B cells, NP-binding and non-binding GC cells and their respective tdTomato^+^ fractions in PBS control or NP-KLH-immunized mice. **c** Frequencies of tdTomato^+^ cells in NP non-binding (NP^*−*^) or NP-specific (NP^+^) GC B cells in NP-KLH-immunized mice. Each symbol represents one mouse, and lines denote mean values. Data were pooled from three independent experiments, with all mice of 3–4 months of age. The *P* value was by a two-tailed unpaired *t*-test. **d** The representative distribution of tdTomato^+^ cells in GCs of PBS control or NP-KLH-immunized mice. Scale bar, 50 μm. **e** Histogram profiles of representative ASC supernatants derived from single NP^–^tdTomato^+^ GC B cells as in Fig. 3a, tested with splenocytes from MD4 mice as target cells. **f** ASI of TCR libraries from indicated T cell populations of NP-KLH-immunized mice, as assayed in NFAT-GFP reporter hybridoma stimulated with WT or class II MHC-deficient B cells (blue) or DCs (red). **g** The protocol for labeling spontaneous GCs prior to LCMV infection. **h** Representative flow-cytometry profiles, showing GCs in B220^+^ B cells, SPPC in total splenocytes and their respective tdTomato^+^ fractions in control or LCMV-infected mice 14 days post infection. **i** Total numbers of tdTomato^+^ GCs and SPPCs in the spleen of control or LCMV-infected mice 7 and 14 days after infection (8 and 15 days after labeling). Each symbol represents one mouse, and lines denote mean values. Data were pooled from two independent experiments, with all mice of 3–4 months of age. *P* values by Mann–Whitney tests. **j** Histogram profiles of staining of B220^+^IgD^+^ B cells or CD4^+^ T cells from FcγRIIB-deficient mice. Primary reagents were sera from control or infected (4-month old) mice, collected 14 days after infection. Mouse IgG1 (0.5 mg/mL) was used as the negative control. **k** MFIs of staining as in **i**. Each symbol represents one mouse serum sample, and lines denote mean values. *P* values by Mann–Whitney tests.

Given the constitutive nature of spontaneous GCs, GCs induced by immunization or infection would likely be intermingled with BDA-reactive GC cells. To test this possibility, we treated the S1PR2-CreERT2;Rosa-Ai14 reporter strain with tamoxifen to label spontaneous GCs before NP-Keyhole Limpet Hemocyanin (KLH) immunization (Fig. 6a). By flow cytometry, tdTomato labeled only spontaneous GCs (80%–90%) already existed before immunization and did not label any NP-specific GC B cells induced after immunization (Fig. 6b, c), demonstrating the specificity of this approach. As shown in Fig. 6d, while sporadic but tightly packed tdTomato^+^EFNB1^+^spontaneous GC clusters were seen in non-immunized mice, large GCs were seen to contain tdTomato^+^ cells 8 days after NP-KLH immunization (29 out of 32 GCs from 8 mice examined in 2 independent experiments). Analysis of single GC B cells using the protocol in Fig. 3a revealed that 3 of 14 tdTomato^+^ cells that did not bind to NP were reactive to surface BDAs (Fig. 6e). Therefore, immunization-induced foreign antigen-reactive GCs merge with pre-existing spontaneous GCs. Using the same approach as described in Fig. 5e, we further constructed α chain libraries for T_FH_ cells, T_FR_ cells, conventional non-T_FH_ activated T cells, and Treg cells isolated from NP-KLH-immunized *Tcrb*^*1D2/1D2*^*Foxp3*^*hCD2/y*^ or *Tcrb*^*1D2/1D2*^*Foxp3*^*hCD2/hCD2*^ mice. As shown in Fig. 6f and Supplementary information, Fig. S10, TCR repertoires of polyclonal T_FH_ cells and T_FR_ cells following immunization still contained specific reactivities toward B cells. Therefore, BDA-reactive B cells, T_FH_ and T_FR_ cells are a regular component in GC responses even after immunization. Interestingly, we notice that ASI of T_FH_ cells isolated from immunized mice is much lower than that of spontaneous T_FH_ cells, ~3 in the former vs ~15 in the latter, consistent with newly generated immunogen-specific T_FH_ cells being dominant in the total T_FH_ population. On the other hand, ASI of T_FR_ cells isolated from immunized mice is quite similar to the ASI of spontaneous T_FR_ cells, ~2 in the former vs ~3 in the latter, indicating that the T_FR_ repertoire is less affected by immunization.

We conducted similar experiments in mice acutely infected with Lymphocytic Choriomeningitis Virus (LCMV) (Fig. 6g, h). First, between the first and the second week after tamoxifen-induced labeling, the number of tdTomato^+^ GC cells was reduced by ~5 folds, whereas the number of tdTomato^+^ SPPCs did not change in control mice (comparing day 8 and 15 post labeling in Fig. 6i). These data suggest that spontaneous GCs rapidly turn over in a few days without significant output to SPPCs, consistent with homeostatic maintenance by the regulatory triad. In infected mice, the number of tdTomato^+^ GCs was comparable to control mice, either on day 8 or 15 after labeling. In striking contrast, the number of tdTomato^+^ SPPCs in infected mice outstripped that in control mice by 10 folds 7 days after infection (8 days after labeling), and the difference was further increased by another factor of ~10 over the second week after labeling. Moreover, this is despite the fact that tdTomato^+^ GCs in infected mice went through similar decay over the same period as in control mice. These data indicate that, following viral infection, mixed-in spontaneous GC components become highly productive in giving rise to SPPCs, and/or their progeny SPPCs are massively expanded. Consistent with accumulation of SPPCs derived from spontaneous GCs, which prominently contain BDA reactivities, we observed a markedly increased serum IgG response to B-cell but not T-cell surface autoantigen, tested with FcγRIIB-deficient naïve B and T cells as the target (Fig. 6j, k). The reason to use FcγRIIB-deficient cells as the target was to ensure no interference from Fc binding by FcγRIIB. These results suggest that flares of such auto-antibody production following episodes of viral infections could increase the possibility of developing overt autoimmunity.

## Discussion

Survival of autoreactive cells in the circulating repertoire, whether anergic or ignorant, poses a risk of triggering autoimmunity, because under the right condition such cells could become fully activated by their autoantigen.^9^ In this sense, the B-cell follicle would provide the right condition to activate BDA-specific B cells that have passed deletion and anergy checkpoints. Because of those selection checkpoints, affinities of BDA-reactive B cells that participate in spontaneous GCs should be sufficiently low. Indeed, affinities of the three FcγRIIB-reactive antibodies (UNW10, K3H12 and W2H5) we identify appear quite low. This is consistent with previous findings that B cells carrying BCRs of extremely low affinities for antigen can be activated and recruited into a GC response,^41^ presumably because antigen can be displayed on the surface of presenting cells to trigger those B cells. In the case of FcγRIIB as a BDA, it is naturally on the surface of densely packed follicular B cells. Our method of using soluble recombinant antibodies to detect BDA reactivities does not fully recapitulate the situation of surface BCRs interacting with surface BDAs on other cells. Despite this limitation, we still find at least 20% in spontaneous GCs reactive to surface BDAs. Therefore, it is likely that surface BDA-reactive B cells account for a larger fraction of the spontaneous GC response. Our results do not exclude the scenario that spontaneous GCs may contain those B cells reactive to microbiota, food, or other environmental antigen.

GCs induced by foreign antigen via vaccination or infection can physically merge with spontaneous GCs. A fraction of GC B cells following immunization cannot demonstrably bind to the immunogen.^36^ It is possible that some of these cells are pre-existing BDA-reactive GCs that are merged with foreign antigen-specific GCs. It will be important in the future to understand full impacts of BDA- and other autoantigen-reactive cells on GC responses to foreign antigen. FcγRIIB-specific spontaneous GC B cells serve as an informative example. Because FcγRIIB inhibits antigen receptor signaling and B cell activation,^42^ either blocking or agonistic anti-FcγRIIB antibodies likely impact on neighboring cells in the GC.

Antigen-specific interactions between T cells and B cells are positive-feedback in nature.^43,44^ After a sufficient number of autoreactive B cells of even a single epitope specificity are activated to form GCs, by virtue of positive-feedback T–B interactions, epitope spreading could perpetuate autoreactive GC responses independent of the clone originally activated.^45^ We speculate BDA-specific cells in spontaneous GCs might represent very early “tolerance-breaking” cells in systemic autoimmunity. Given the obvious danger, it is counterintuitive that the immune system permits BDA-reactive B cells to develop into spontaneous GCs. Perhaps it results from evolutionary necessities. The benefit of having follicular organization of packed B cells in the secondary lymphoid organs, presumably to facilitate rapid responses to foreign antigen, probably outweighs the danger of having a tinder for autoimmunity that would require contributions of additional risk factors to develop full-blown diseases much later in life. In fact, gauged by surface BDA-specific antibodies in serum, the rate of seral conversion among individuals and the titer (staining intensity) in positive individuals gradually increase as mice normally age. Therefore, without other autoimmune-prone defects in play, BDA reactivities in spontaneous GCs do not automatically lead to overt diseases. In addition, the risk of full-fledged autoimmune diseases could also be tempered by SHM. In an adoptive transfer model, anergic autoreactive B cells are shown to be activated by cross-reactive foreign antigen to form GCs and then mutate away from self specificities to become reactive exclusively to the foreign antigen.^46,47^ On the other hand, we also speculate that spontaneous GCs may serve to facilitate GC responses to foreign antigen in the same lymphoid organs.

The fact that spontaneous T_FH_ cells are enriched with BDA specificities is consistent with the significant contribution of BDA-specific B cells to spontaneous GCs. The fact that same TCRs (e.g., 10 × 1 or 10 × 2) appear in both spontaneous T_FH_ and T_FR_ cells agrees well with the known overlap of TCR repertoires of regulatory and conventional helper T cells.^48^ Naïve precursors to BDA-specific T_FH_ cells are expected to be continuously generated as far as thymic output is available, and these T_FH_ cells would not fade away as far as spontaneous GCs persist. Because BDAs are by definition presented by all B cells, BDA-specific T_FH_ cells could in principle help GC B cells of any specificity and may thus serve as a trophic support provider for foreign antigen-induced GC reaction. Consistent with this possibility, we observe BDA-reactive TCR specificities in the T_FH_ population during immunization-induced GC responses. On the other hand, because immunization also induces foreign antigen-specific T_FH_ cells that specifically help foreign antigen-specific GC but not BDA-specific GC cells, a natural advantage of the former in positive selection would be guaranteed. A recent TCR repertoire analysis suggests that T_FH_ cells following immunization harbor diverse bystander clones unspecific to immunogen,^49^ potentially reflecting participation by BDA-reactive T_FH_ cells. Future studies are required to investigate how BDA-specific T_FH_ cells affect affinity maturation and post-GC development of memory B cells and plasma cells of foreign antigen specificities. How these helper cells are initially primed by dendritic cells and/or B cells directly would also need future investigation.

As follicular autoantigen, BDAs are essentially unlimited in supply. However, spontaneous GCs do not become as big as immunization- or infection-induced GCs, even though they gradually grow as animals age. An important difference appears to be in the T_FR_-to-T_FH_ ratio, which is much larger in spontaneous GCs than in immunization- or infection-induced GCs. Similar to BDA-reactive spontaneous T_FH_ cells, BDA-specific T_FR_ cells could in principle interact with any B cells in an antigen-specific manner, and BDA-reactive B cells would be subjected to suppression in particular by these T_FR_ cells. This latter notion is supported by the observation that, when T_FR_ cells are reduced, the frequency of surface BDA-reactive B cells is increased.

Under the steady state without deliberate immunization or infection, we observe four effects of T_FR_ insufficiency: increased overall spontaneous GCs, increased overall plasma cells, increased isotype-switched cells in GCs and plasma cells, and increased BDA-specific autoantibodies and antibodies of other self specificities (e.g., dsDNA). One or another of these aspects has previously been observed in models of immunization or infection but rarely all four in one model.^27–29,50–54^ It is tempting to speculate that the reason for such variation is because T_FR_-mediated suppression may be primarily and most efficiently targeted toward the BDA-reactive spontaneous GC component, which, to various extent, co-exists within immunization- or infection-induced GCs. The fact that LCMV infection leads to massive accumulation of spontaneous GC-derived plasma cells, including those reactive to surface BDAs, is presumably because the balance between T_FH_ help and T_FR_ suppression is tilted toward the former by the inflammatory environment during viral infection. It remains to be determined as to whether T_FR_-mediated regulation is through direct inhibition of B cells or indirect inhibition by denying them access to T_FH_ help or both. Details of tripartite interactions among B cells, T_FH_ cells and T_FR_ cells notwithstanding, the BDA-specific triad appears perpetuated spontaneously in a bona fide T-dependent GC response under the homeostatic state, also participating as a regular component in immunization- and infection-induced responses.

## Materials and methods

### Mice

B6 (Jax 664), HEL-specific Ig-transgenic MD4 (Jax 2595), Foxp3IRES-GFP knock-in reporter (Jax 6772), green fluorescent protein (GFP)-expressing (Jax 4353), RosaLSL-tdTomato (Ai14, Jax 7914), class II MHC-deficient (Jax 3584), and FcγRIIB-deficient (Jax 2848) mice were originally from the Jackson Laboratory. Germ-free B6 mice were obtained from Laboratory Animal Resource Center at Tsinghua University. The Foxp3-GFP-hCre bacterial artificial chromosome transgenic B6 mice (called “Foxp3-cre”) was previously reported.^33^ *Sh2d1a*^*−/−*^ (SAP-deficient), *Bcl6*fl/fl, S1PR2ERT2cre and Foxp3hCD2 TCRβ-1D2 B6 mice were kindly provided as a gift by Drs. P. Shwartzberg,^55^ T. Takemori,^56^ Dr. T. Kurosaki^57^ and Dr. S. Hori, respectively. All relevant alleles were maintained on the B6 background. Age- and sex-matched mice were used in all experiments. All mice were maintained under specific pathogen-free conditions unless indicated otherwise and used in accordance of governmental and institutional guidelines for animal welfare.

### Spontaneous GC labeling, immunization and infection

For certain experiments, S1PR2ERT2cre;RosaLSL-tdTomato mice were given 2 mg tamoxifen by gavage every day for 4 consecutive days. Forty eight h after the last tamoxifen treatment, those mice were immunized intraperitoneally with 50 μg NP-KLH (Biosearch Technologies) mixed with 1 μg lipopolysaccharide (LPS) (Sigma) in alum (Thermo Scientific). Some mice were infected intraperitoneally with 5 × 10^5^ PFU LCMV Armstrong strain.

### CD40L blockade in vivo

Female B6 mice (5-month-old) were intravenously injected with 200 μg blocking anti-CD40L (MR1, Biolegend) or isotype antibody (HTK888, Biolegend) in 300 μL PBS per mouse on day 0 and day 2. On day 4, mice were sacrificed to determine spontaneous GC formation.

### Flow cytometry

Spleen and bone marrow cells were incubated in MACS buffer PBS supplemented with 1% fetal bovine serum (FBS) and 5 mM ethylenediaminetetraacetic acid containing Fc blocker (2.4G2 hybridoma supernatant) for 20 min before stained with indicated reagents. The staining reagents used include: BV421-anti-CD4 (GK 1.5), PE-Cy7-anti-CD95 (Jo2), APC-anti-CD138 (281-2), v450-anti-LY6G/C (RB6-8C5), PE-streptavidin, APC-anti-FOXP3 (FJK-16s), PE-anti-IgG1 (A85-1), PE-anti-IgMa (DS-1) from BD Biosciences, eFlour450-GL7 (GL-7), FITC-anti-IgD (11-26 C), AF700-anti-CD44 (IM7e) from eBioscience, PE-Cy7-anti-PD-1 (RMP1-30), APC-Cy7-anti-B220 (RA3-6B2), APC-anti-TCRβ chain (H57-597) from Biolegend, biotinylated or PE-anti-CXCR5 (REA215) from Miltenyi Biotec, AF488-AffiniPure Goat anti-mouse IgG (code: 115-545-071). Dead cells were excluded from analysis by staining with 7-AAD (Biotium) or by using the Zombie Yellow Fixable Viability kit (Biolegend). Surface staining was done on ice with primary reagents incubated for 30 or 90 min, followed by secondary reagents for 30 min with washes in-between. For FOXP3 staining, the FOXP3/Transcription Factor Staining Buffer Set (eBioscience) was used according to the manufacturer’s protocol. For intracellular IgMa staining of plasma cells, the CytoFix/Perm kit (BD Biosciences) was used according to the manufacturer’s protocol. All flow cytometry data were collected on a LSR II or an Aira III (BD Biosciences) and analyzed with FlowJo software (TreeStar).

### Immunohistochemistry

Spleens were fixed with periodate-lysine-paraformaldehyde fixative for 8–12 h and then dehydrated in 30% sucrose overnight before being embedded in OCT (Sakura). OCT-embedded tissues were cut into 16 μm sections, blocked in 0.1 M Tris-HCl buffer with 0.3% Triton and 1% FBS for 1 h. Sections were then stained with PE-anti-CD4 (GK1.5, Biolegend), eFlour450-anti-IgD (11-26c, eBiosicence) and polyclonal goat-anti-mouse EFNB1 (R&D) to label the T-cell zone, follicles and GCs, respectively. AlexaFluor 647-labeled donkey anti-goat IgG (Invitrogen) was used as the secondary antibody to reveal anti-EFNB1 staining. For certain experiments involving detection of Treg cells using Foxp3IRES-GFP mice, to better reveal FOXP3-expressing cells, sections were stained additionally for GFP with a polyclonal rabbit-anti-GFP (Abcam), revealed by a donkey-anti-rabbit IgG labeled with AlexaFluor 488 (Invitrogen). Tissue sections were mounted with the ProlongGold Antifade reagent (Invitrogen) and examined with an Olympus FV1000 upright microscope. Imaging data were analyzed with Imaris 7.2 (Bitplane) and ImageJ 1.46r (NIH).

### Culture of single GC B cells

NIH-3T3 feeder cells that stably express CD40L and BAFF were seeded into 96-well plates at 15,000 cells per well in 100 μL complete RPMI-1640 one day before receiving B cells. On day 0, single FAS^+^GL7^+^ GC B cell was sorted directly into these feeder wells, supplemented with recombinant mouse IL-4 (2 ng/mL, Peprotech) and IL-21 (2.5 ng/mL, Peprotech). On day 2, 50 μL culture medium was removed and each well was replenished with 100 μL fresh culture medium containing IL-21 (2.5 ng/mL, Peprotech). From day 3 to 8, 100 μL old medium was replaced with 100 μL fresh medium containing IL-21 (2.5 ng/mL, Peprotech) for each well every day. On day 9 and 10, culture supernatants were harvested (antibody-secreting cell supernatants) and used as cytometry staining or ELISA detection reagent for subsequent assays. These supernatants typically contained ~10 μg/mL total mouse IgG by ELISA. Single-cell cultures in plates were frozen stored at –80 °C until subsequent use for cloning of Ig-coding sequences using a published protocol.^58^

### Production of recombinant whole-molecule and F(ab)_2_ antibodies

Coding sequences of the light chain and the variable region of the heavy chain for each single GC B-cell culture were amplified and inserted into the pRK5 expression plasmid, which for heavy chain already contains the coding sequence for mouse IgG1 (mIgG1) Fc. Equal amounts of light chain- and heavy chain-coding vector DNA was transiently transfected into 293 F cells (R790-07, Invitrogen) with polyethyleneimine, when cells were grown to 80% confluency. Cells were grown in FreeStyle™ 293 Expression Medium (12338-018, Invitrogen). Supernatants were harvested 6 days after transfection. For 50 mL 293 F supernatants, 50 μL rProtein A beads (SA012005, SMART lifesciences) were used to absorb recombinant antibodies overnight on a rocker at 4 °C. After two rounds of washing with PBS, antibodies were eluted with 0.05 M Glycine (pH 2.8). Neutralizing buffer (1 M Tris-HCl pH 8.0) was then added to eluates for pH neutralization. Amicon® Ultra-15 centrifugal filters, 50 KD (UFC9050, Millipore) were used to exchange buffer to PBS. Concentrations of recombinant IgG1 antibody were measured by NanoDrop (Thermo scientific). For F(ab)’_2_ production, the coding sequence for mIgG1 Fc region in the pRK5 expression plasmid was replaced with the coding sequence for mouse mIgG1 CH1 and hinge region followed by 6× His tag. After co-transfection of light chain- and heavy chain-coding vector DNA into 293 F cells and expression, F(ab)’_2_ were purified from supernatant through Ni-chelating affinity chromatography as ~100 kDa components by gel filtration.

### Cell isolation, culture and retroviral transduction

Splenic B cells were isolated by CD19 Microbeads (Miltenyi Biotec). For certain experiments, isolated B cells were cultured in complete RPMI-1640 with 1 μg/mL LPS (Sigma) for 2 days before being used for the conjugate assay or hybridoma reporter assay. For other experiments, B cells cultured in complete RPMI-1640 with 10 μg/mL F(ab’)_2_ anti-mouse IgM (Jackson Immunosearch) and 10 μg/mL anti-mouse CD40 (FGK45, BioXcell) before retroviral transduction. Splenic DCs were isolated by CD11c Microbeads (Miltenyi Biotec) from single cell suspension made after digestion with 40 μg/mL collagenase D and 20 μg/mL DNase I (Roche) at 37 °C for 30 min. Isolated DCs were either immediately used in the conjugate assay or stimulated in complete RPMI-1640 with 8 μg/mL LPS (Sigma) for 12 h before being used in the hybridoma reporter assay. Splenic CD4^+^ T cells were isolated from CD4 Microbeads (Miltenyi Biotec) and, when applicable, activated with plate-bound anti-CD3 and anti-CD28 (BioXcell). Hybridoma cells were cultured in complete DMEM for passaging or in complete RPMI-1640 for co-culture with B cells or DCs. For retroviral transduction, all cDNA constructs were cloned into a murine stem cell virus (MSCV)-based retroviral vector. Retrovirus was packaged with the Plate-E system, as described previously.^59^ After retroviral infection, B cells or T cells were cultured in complete RPMI-1640 for 2 days before subsequent experiments.

### T cell–APC conjugate assay

Polyclonal T cells from GFP-expressing mice were retrovirally transduced with indicated TCR constructs. Splenic B cells that were activated with 1 μg/mL LPS (Sigma) for 2 days or freshly isolated splenic DCs (CD11c^+^) were stained with 50 mM CellTrace Violet (Invitrogen) at 37 °C for 15 min. T cells (2.5 × 10^5^ per tube) were spun down at 300× *g* together with B cells (5 × 10^5^ per tube) or DCs (2.5 × 10^5^ per tube) and then incubated at 37 °C for 30 min. After vortexing for 30 s, frequencies of T–B or T–DC conjugates were enumerated by flow cytometry as previously described.^60^

### Detection of reactivities to B cell surface autoantigen

Splenocytes (2 × 10^5^ per sample) from MD4 or FcγRIIB-deficient mice were incubated on ice for 1.5 h with supernatants from single-GC B cell culture or 20 μg/mL recombinant mIgG1 antibodies cloned as above. After washing in PBS, cells were stained with FITC-anti-mouse IgG antibody (culture supernatants as the primary) or PE-anti-mouse IgG1 antibody (recombinant mIgG1 as the primary) in addition to rat monoclonal antibodies against mouse B220 and CD4 to reveal B cells, T cells and non-B/T cells in the target splenocytes. For certain experiments to demonstrate specific binding of recombinant mIgG1 antibodies to the FcγRIIB molecule, 2 × 10^5^ splenocytes of WT or FcγRIIB-deficient mice or 2 × 10^5^ B cells overexpressing FcγRIIB were used as the target. When indicated, the 2.4G2 hybridoma supernatant was used to pre-treat the target cells. Stained cells were analyzed by flow cytometry.

### FcγRIIB-specific ELISA

The extracellular domain of FcγRIIB was expressed as recombinant protein and used at 2 μg/mL in PBS to coat ELISA plates at 4 °C overnight. Plates were then blocked with 5% BSA (Cat# 180728, MP Biomedicals) and incubated with diluted recombinant F(ab)’_2_ antibodies at room temperature for 2 h. After washings with PBST, plates were further incubated with horseradish peroxidase (HRP)-conjugated anti-mouse IgG F(ab)’_2_ (Goat F(ab)’_2_, 1:10,000; Jackson ImmunoResearch, Cat# 115-036-072) at room temperature for 2 h before development with TMB substrate set (Cat# 421101, Biolegend). The chromogenic reaction was stopped by addition of 1 M HCL, and optical density (OD) was read on an iMark plate reader (Bio-rad).

### Barcoded anti-CD3 antibody for TCR sequencing

To make barcoded anti-CD3, 40 μg antibody were exchanged into PBS buffer and concentrated into 50 μL with the Amicon Ultra 0.5 mL 30 kDa MWCO centrifugal filter (Millipore) by two additions of 500 μL PBS and centrifugation at 14,000× *g* for 10 min at 4 °C; 24 μL Dibenzocyclooctyne-PEG4-N-hydroxysuccinimidyl ester (DBCO-PEG4-NHS) were then added to the antibody solution for a 2 h incubation at 4 °C with rotation. Residual DBCO reagent was removed by 5× washings with 300 μL PBS buffer each using the 30 kDa filter. DBCO antibodies in 60 μL were then divided into 8 tubes, each of which was mixed with a different 5′-azide barcoded DNA oligo (CGGAGATGTGTATAAGAGACAG-15nt barcode-CCCATATAAGA*A*A; * represents phosphorothioate, ordered from Sangon Biotech). The mixture was incubated at 4 °C overnight with rotation. Residual DNA oligos were removed by 5× washings with 300 μL PBS buffer each using the 30 kDa filter.

### TCR and BCR sequencing by 10× Genomics

For some TCR sequencing experiments, CD4^+^ T cells of indicated subsets were isolated by MACS beads (Miltenyi Biotec) from a pool of 8 Foxp3-IRES-GFP reporter mice. For some other experiments, CD4^+^ T cells from individual Foxp3-IRES-GFP donor mice were isolated separately and labeled with different DNA-barcoded ef660-anti-CD3 antibodies. After proper surface staining, total splenic CD4^+^ T cells were subjected to sorting for T_H_ (CD4^+^CD44^+^CXCR5^–^PD-1^–^Foxp3^–^), Treg (CD4^+^CD44^+^CXCR5^–^PD-1^–^Foxp3^+^), T_FH_ (CD4^+^CD44^+^CXCR5^+^PD-1^+^Foxp3^–^), and T_FR_ (CD4^+^CD44^+^CXCR5^+^PD-1^+^Foxp3^+^) cells. For BCR sequencing experiments, GC and plasma cells from T_FR_-sufficient and -insufficient mice were enriched by anti-GL7 or anti-CD138 MACS beads. After proper surface staining, GC (B220^+^FAS^+^GL7^+^) and plasma cells (B220^low^CD138^+^) were sort-purified by flow cytometry.

T or B cell samples were resuspended to a concentration of ~800 cells/μL. The 10× Genomics Chromium Next GEM Single Cell 5′ Reagent Kits v2 standard protocol was followed. The targeted cell recovery number was 8000. To retrieve barcodes from individual mice, 5 μL of the 0.2 μM barcode amplification primer (5′-GTCTCGTGGGCTCGGAGATGTGTATAAGAGACAG-3′) was added to the cDNA amplification reaction, and amplified barcodes were separated from the cDNA by size selection DNA purification using the AMPure XP beads (Beckman Coulter), and sequencing adapter was then added to the barcode amplified products by index PCR. Each sample library was sequenced to obtain 40 Gb raw data by illumina novaseq PE150. TCR and BCR full-length sequence was demultiplexed and assembled by Cellranger 5.0.0 with standard TCR and BCR reference provided by 10×X. The TCR and BCR full-length data were filtered by only one paired TCRα and TCRβ for one cell (for BCR, only one paired heavy chain and light chain for one cell).

### Construction of TCRα chain library

To construct TCRα chain library for spontaneous T_FH_ cells, splenic CD4^+^ T cells were isolated by MACS beads (Miltenyi Biotec) from 13 *Foxp3*^*hCD2/y*^*Tcrb*^*1D2/1D2*^ male and 13 *Foxp3*^*hCD2/hCD2*^*Tcrb*^*1D2/1D2*^ female 2- to 3-month-old mice. Subsequently, a total of 6 × 10^5^ T_H_ (CD4^+^CD44^+^CXCR5^–^PD-1^–^hCD2^–^), 2.5 × 10^5^ Treg (CD4^+^CD44^+^CXCR5^–^PD-1^–^hCD2^+^), 6 × 10^5^ T_FH_ (CD4^+^CD44^+^CXCR5^+^PD-1^+^hCD2^–^), and 1.7 × 10^5^ T_FR_ (CD4^+^CD44^+^CXCR5^+^PD-1^+^hCD2^+^) cells were sort-purified by Aira III sorter (BD Biosciences). To construct TCRα chain library for T_FH_ cells following immunization, splenic CD4^+^ T cells were isolated by MACS beads from 5 *Foxp3*^*hCD2/y*^*Tcrb*^*1D2/1D2*^ male and 4 *Foxp3*^*hCD2/hCD2*^*Tcrb*^*1D2/1D2*^ female 2- to 3-month-old mice 13 days post NP-KLH immunization. Subsequently, a total of 4 × 10^5^ T_H_, 1.6 × 10^5^ Treg, 3.5 × 10^5^ T_FH_, and 1 × 10^5^ T_FR_ cells were sort-purified by Aira III sorter. RNA from indicated T cell populations was extracted with RNA extraction Kit (R4111-02, Magan). TCRα cDNA library amplification was done as previously reported.^61^ The libraries were cloned into an MSCV vector that also encodes red fluorescent protein (RFP) by ClonExpress II one step cloning kit (C112, Vazyme). The TCRα library of each T cell population was derived from at least 10^5^ *Escherichia coli* (*E.coli*) clones developed on lysogeny broth (LB) plates. Retroviral TCRα library was packaged with the Plate-E system.

### mhCD4-NFAT-GFP and 1D2β-mhCD4-NFAT-GFP hybridoma reporter assays

A mutated version of human CD4 that carries Q40Y and T45W replacements (mhCD4) increases affinity for class II MHC molecule.^62^ We cloned mhCD4 into an MSCV-based retroviral vector that also encodes mAmetrine. NFAT-GFP hybridoma cells^39^ were transduced with MSCV-mhCD4-mAmetrine, and mAmetrine^+^cells were sort-purified to generate mhCD4-NFAT-GFP hybridoma line. As previously reported,^63^ mhCD4-NFAT-GFP is more sensitive to TCR stimulation by class II MHC-presented antigen than the original NFAT-GFP hybridoma. We cloned the β chain-coding sequence of 1D2 TCR into an MSCV vector that also encodes mouse CD4 (mCD4). mhCD4-NFAT-GFP hybridoma were transduced with this MSCV-1D2β-mCD4, and mCD4^+^ cells were sort-purified to generate 1D2β-mhCD4-NFAT-GFP hybridoma.

To establish hybridoma cells expressing 10 × 1, 10 × 2, Tfh13, and Tfh14 TCR, we cloned the α and β chains connected by a 2 A peptide into an MSCV vector that also encodes mouse CD4 (mCD4). mhCD4-NFAT-GFP hybridoma were transduced with MSCV-TCRα-P2A-TCRβ-mCD4 constructs coding for 10 × 1, 10 × 2, Tfh13, and Tfh14, respectively, and surface mCD4^+^TCRb^+^ cells were sort-purified.

To establish hybridoma cells expressing TCR repertoires derived from different CD4 T cell subsets (T_H_, Treg, T_FH_, and T_FR_), 1D2β-mhCD4-NFAT-GFP hybridoma were transduced with corresponding TCRα libraries carried in an MSCV-TCRα-RFP vector, and surface RFP^+^ TCRb^+^ cells were sorted.

To measure reactivities of TCRs toward B cell- or DC-presented antigen, TCR-reconstituted hybridomas (3.5 × 10^4^ cells) were co-cultured with splenic B cells (7 × 10^5^ cells) that were pre-activated by 1 μg/mL LPS (Sigma) for 2 days or with splenic DCs (3.5 × 10^5^ cells) that were pre-activated by 8 μg/mL LPS (Sigma) for 12 h. To compare abilities of B cells and DCs to present exogenous antigen to T cells, a titrated series of OVA peptide were added to co-culture of B cells and DCs activated as above and hybridomas transduced by the OT-II TCR. The hybridoma co-culture was incubated in 96-well round bottom plates for 22–24 h before GFP expression by hybridoma cells was measured by flow cytometry.

### Detection of autoantibodies in sera

To detect antibodies reactive to surface autoantigen on B cells, splenocytes from FcγRIIB-deficient mice were used as target cells. Sera obtained from 15- to 17-month-old T_FR_-sufficient or -insufficient mice, LCMV-infected (day 14) or control mice were used as the primary reagent to stain these cells at 1:2 dilution in PBS for 1.5 h. Polyclonal mouse IgG1 was used at 0.5 mg/mL as the negative control. Staining was revealed with PE-conjugated anti-mouse IgG1 antibody. B cell targets in splenocytes were identified by B220^+^IgD^+^ and CD4 T cells were identified by CD4 staining.

### Statistical analyses

Statistical analyses and graphing were done in Prism (Graphpad). Two-tailed unpaired Student’s *t* -tests were used to compare end-point means of different groups unless indicated otherwise. Mann–Whitney tests were used to analyze data that exhibited non-uniform, skewed distributions. Fisher’s exact tests were used for analyzing categorical data. To analyze surviving curves, log-rank (Mantel–Cox) tests were used.

### Supplementary information

Supplementary information, Fig. S1

Supplementary information, Fig. S2

Supplementary information, Fig. S3

Supplementary information, Fig. S4

Supplementary information, Fig. S5

Supplementary information, Fig. S6

Supplementary information, Fig. S7

Supplementary information, Fig. S8

Supplementary information, Fig. S9

Supplementary information, Fig. S10

Supplementary information, Table S1

Supplementary information, Table S2

## Data Availability

All data generated and/or analyzed during the current study are available from the corresponding author upon reasonable request.

## References

[1] Kara EE, Nussenzweig MC (2018). Redemption for self-reactive antibodies. Science.

[2] Gay D, Saunders T, Camper S, Weigert M (1993). Receptor editing: an approach by autoreactive B cells to escape tolerance. J. Exp. Med..

[3] Tiegs SL, Russell DM, Nemazee D (1993). Receptor editing in self-reactive bone marrow B cells. J. Exp. Med..

[4] Nemazee DA, Burki K (1989). Clonal deletion of B lymphocytes in a transgenic mouse bearing anti-MHC class I antibody genes. Nature.

[5] Goodnow CC (1988). Altered immunoglobulin expression and functional silencing of self-reactive B lymphocytes in transgenic mice. Nature.

[6] King LB, Monroe JG (2000). Immunobiology of the immature B cell: plasticity in the B-cell antigen receptor-induced response fine tunes negative selection. Immunol. Rev..

[7] Wardemann H (2003). Predominant autoantibody production by early human B cell precursors. Science.

[8] Aplin BD (2003). Tolerance through indifference: autoreactive B cells to the nuclear antigen La show no evidence of tolerance in a transgenic model. J. Immunol..

[9] Shlomchik MJ (2008). Sites and stages of autoreactive B cell activation and regulation. Immunity.

[10] Cyster JG (2005). Chemokines, sphingosine-1-phosphate, and cell migration in secondary lymphoid organs. Annu. Rev. Immunol..

[11] Garside P (1998). Visualization of specific B and T lymphocyte interactions in the lymph node. Science.

[12] Cyster JG (2010). B cell follicles and antigen encounters of the third kind. Nat. Immunol..

[13] Brink R, Phan TG (2018). Self-reactive B cells in the germinal center reaction. Annu. Rev. Immunol..

[14] Legoux FP (2015). CD4+ T cell tolerance to tissue-restricted self antigens is mediated by antigen-specific regulatory T cells rather than deletion. Immunity.

[15] Yu W (2015). Clonal deletion prunes but does not eliminate self-specific alphabeta CD8(+) T lymphocytes. Immunity.

[16] Victora GD, Nussenzweig MC (2012). Germinal centers. Annu. Rev. Immunol..

[17] MacLennan IC (1994). Germinal centers. Annu. Rev. Immunol..

[18] Luzina IG (2001). Spontaneous formation of germinal centers in autoimmune mice. J. Leukoc. Biol..

[19] Soni C (2014). B cell-intrinsic TLR7 signaling is essential for the development of spontaneous germinal centers. J. Immunol..

[20] Laidlaw BJ (2017). The Eph-related tyrosine kinase ligand Ephrin-B1 marks germinal center and memory precursor B cells. J. Exp. Med..

[21] Lu P, Shih C, Qi H (2017). Ephrin B1-mediated repulsion and signaling control germinal center T cell territoriality and function. Science.

[22] Qi H, Cannons JL, Klauschen F, Schwartzberg PL, Germain RN (2008). SAP-controlled T-B cell interactions underlie germinal centre formation. Nature.

[23] Crotty S, Kersh EN, Cannons J, Schwartzberg PL, Ahmed R (2003). SAP is required for generating long-term humoral immunity. Nature.

[24] Yusuf I (2010). Germinal center T follicular helper cell IL-4 production is dependent on signaling lymphocytic activation molecule receptor (CD150). J. Immunol..

[25] Germain RN (2012). Maintaining system homeostasis: the third law of Newtonian immunology. Nat. Immunol..

[26] Josefowicz SZ, Lu LF, Rudensky AY (2012). Regulatory T cells: mechanisms of differentiation and function. Annu. Rev. Immunol..

[27] Wollenberg I (2011). Regulation of the germinal center reaction by Foxp3+ follicular regulatory T cells. J. Immunol..

[28] Chung Y (2011). Follicular regulatory T cells expressing Foxp3 and Bcl-6 suppress germinal center reactions. Nat. Med..

[29] Linterman MA (2011). Foxp3+ follicular regulatory T cells control the germinal center response. Nat. Med..

[30] Johnston RJ (2009). Bcl6 and Blimp-1 are reciprocal and antagonistic regulators of T follicular helper cell differentiation. Science.

[31] Nurieva RI (2009). Bcl6 mediates the development of T follicular helper cells. Science.

[32] Yu D (2009). The transcriptional repressor Bcl-6 directs T follicular helper cell lineage commitment. Immunity.

[33] Zhou X (2008). Selective miRNA disruption in T reg cells leads to uncontrolled autoimmunity. J. Exp. Med..

[34] Lightman SM, Utley A, Lee KP (2019). Survival of long-lived plasma cells (LLPC): piecing together the puzzle. Front. Immunol..

[35] Nojima T (2011). In-vitro derived germinal centre B cells differentially generate memory B or plasma cells in vivo. Nat. Commun..

[36] Kuraoka M (2016). Complex antigens drive permissive clonal selection in germinal centers. Immunity.

[37] Nimmerjahn F, Ravetch JV (2008). Fcgamma receptors as regulators of immune responses. Nat. Rev. Immunol..

[38] Qin D (2000). Fc gamma receptor IIB on follicular dendritic cells regulates the B cell recall response. J. Immunol..

[39] Ise W (2010). CTLA-4 suppresses the pathogenicity of self antigen-specific T cells by cell-intrinsic and cell-extrinsic mechanisms. Nat. Immunol..

[40] Maceiras AR (2017). T follicular helper and T follicular regulatory cells have different TCR specificity. Nat. Commun..

[41] Dal Porto JM, Haberman AM, Kelsoe G, Shlomchik MJ (2002). Very low affinity B cells form germinal centers, become memory B cells, and participate in secondary immune responses when higher affinity competition is reduced. J. Exp. Med..

[42] Smith KG, Clatworthy MR (2010). FcgammaRIIB in autoimmunity and infection: evolutionary and therapeutic implications. Nat. Rev. Immunol..

[43] Qi H (2012). From SAP-less T cells to helpless B cells and back: dynamic T-B cell interactions underlie germinal center development and function. Immunol. Rev..

[44] Shlomchik MJ (2009). Activating systemic autoimmunity: B’s, T’s, and tolls. Curr. Opin. Immunol..

[45] Degn SE (2017). Clonal evolution of autoreactive germinal centers. Cell.

[46] Sabouri Z (2014). Redemption of autoantibodies on anergic B cells by variable-region glycosylation and mutation away from self-reactivity. Proc. Natl. Acad. Sci. USA.

[47] Burnett DL (2018). Germinal center antibody mutation trajectories are determined by rapid self/foreign discrimination. Science.

[48] Hogquist KA, Jameson SC (2014). The self-obsession of T cells: how TCR signaling thresholds affect fate ‘decisions’ and effector function. Nat. Immunol..

[49] Ritvo PG (2018). High-resolution repertoire analysis reveals a major bystander activation of Tfh and Tfr cells. Proc. Natl. Acad. Sci. USA.

[50] Botta D (2017). Dynamic regulation of T follicular regulatory cell responses by interleukin 2 during influenza infection. Nat. Immunol..

[51] Wu H (2016). Follicular regulatory T cells repress cytokine production by follicular helper T cells and optimize IgG responses in mice. Eur. J. Immunol..

[52] Lu Y (2021). CD4+ follicular regulatory T cells optimize the influenza virus-specific B cell response. J. Exp. Med..

[53] Laidlaw BJ (2017). Interleukin-10 from CD4(+) follicular regulatory T cells promotes the germinal center response. Sci. Immunol.

[54] Gonzalez-Figueroa P (2021). Follicular regulatory T cells produce neuritin to regulate B cells. Cell.

[55] Czar MJ (2001). Altered lymphocyte responses and cytokine production in mice deficient in the X-linked lymphoproliferative disease gene SH2D1A/DSHP/SAP. Proc. Natl. Acad. Sci. USA.

[56] Kaji T (2012). Distinct cellular pathways select germline-encoded and somatically mutated antibodies into immunological memory. J. Exp. Med..

[57] Shinnakasu R (2016). Regulated selection of germinal-center cells into the memory B cell compartment. Nat. Immunol..

[58] Tiller T, Busse CE, Wardemann H (2009). Cloning and expression of murine Ig genes from single B cells. J. Immunol. Methods.

[59] Xu H (2013). Follicular T-helper cell recruitment governed by bystander B cells and ICOS-driven motility. Nature.

[60] Chu C (2014). SAP-regulated T Cell-APC adhesion and ligation-dependent and -independent Ly108-CD3zeta interactions. J. Immunol..

[61] Ise W (2010). CTLA-4 suppresses the pathogenicity of self antigen-specific T cells by cell-intrinsic and cell-extrinsic mechanisms. Nat. Immunol..

[62] Wang XX (2011). Affinity maturation of human CD4 by yeast surface display and crystal structure of a CD4-HLA-DR1 complex. Proc. Natl. Acad. Sci. USA.

[63] Williams T (2018). Development of T cell lines sensitive to antigen stimulation. J. Immunol. Methods.

