# Supplementary material for: B cell-reactive triad of B cells, follicular helper and regulatory T cells at homeostasis
Source: Cell Res. 2024 Feb 7;34(4):295–308. doi: 10.1038/s41422-024-00929-0 (PMC10978943; doi:10.1038/s41422-024-00929-0)
Supplement: Supplementary file 1 — Supplementary information, Fig. S1 [file 41422_2024_929_MOESM1_ESM.pdf]

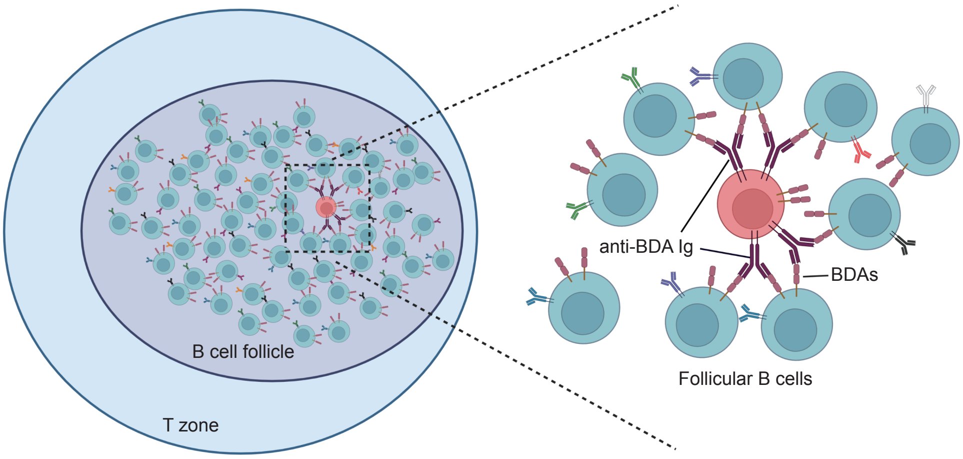

Supplementary information, Fig. S1 A hypothetical model of B cells activated by B cell-derived autoantigen on the surface of follicular B cells.

B cells carrying BCRs specific to B cell-derived autoantigen (BDAs; shown in dark red) may pass clonal deletion and anergy checkpoints to reach the B cell follicle, in which the densely populated B cells provide surface BDA in a form sufficiently stimulatory to activate BDA-specific B cells to form GCs under the homeostatic condition.
