# Supplementary material for: B cell-reactive triad of B cells, follicular helper and regulatory T cells at homeostasis
Source: Cell Res. 2024 Feb 7;34(4):295–308. doi: 10.1038/s41422-024-00929-0 (PMC10978943; doi:10.1038/s41422-024-00929-0)
Supplement: Supplementary file 2 — Supplementary information, Fig. S2 [file 41422_2024_929_MOESM2_ESM.pdf]

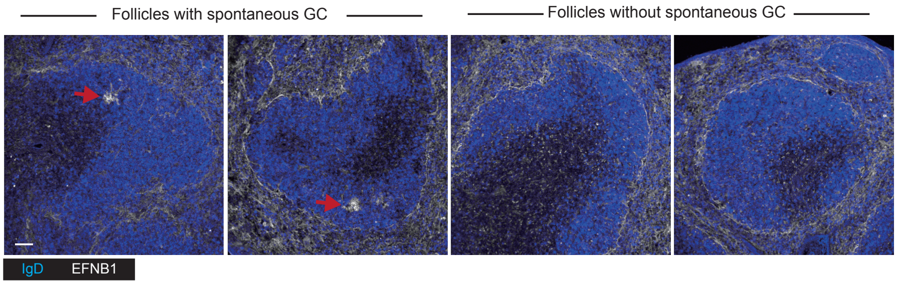

Supplementary information, Fig. S2 Spontaneous GCs in follicles.

Representative images of follicles with visible spontaneous GC clusters or follicles without. Data were obtained from 2- to 3-month-old B6 mice. Scale bar, 50 μm.
