# Supplementary material for: B cell-reactive triad of B cells, follicular helper and regulatory T cells at homeostasis
Source: Cell Res. 2024 Feb 7;34(4):295–308. doi: 10.1038/s41422-024-00929-0 (PMC10978943; doi:10.1038/s41422-024-00929-0)
Supplement: Supplementary file 3 — Supplementary information, Fig. S3 [file 41422_2024_929_MOESM3_ESM.pdf]

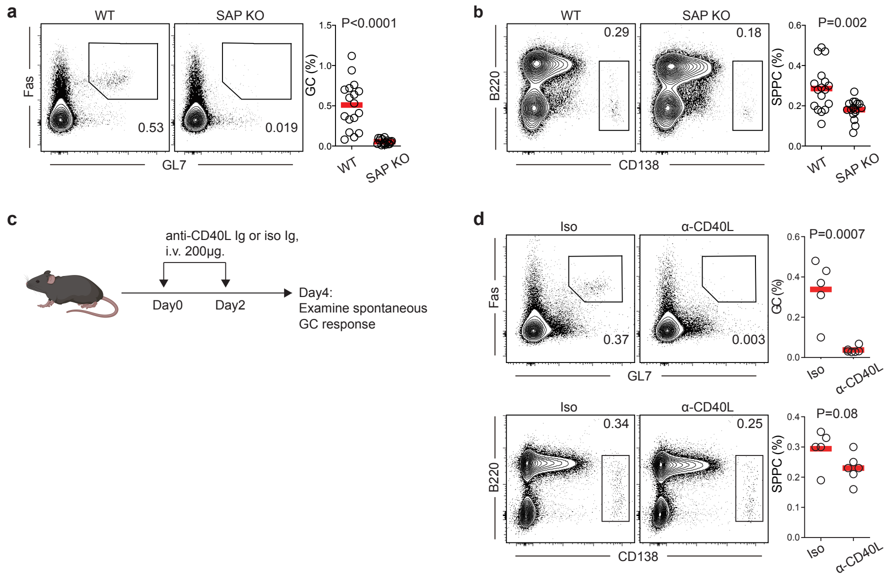

Supplementary information, Fig. S3 Spontaneous GCs require conventional T-cell help.

a-b Spontaneous GCs in wildtype and SAP-deficient B6 mice of 3 to 4 months of age. Representative contour plots and summary statistics of GC (**a**) and SPPC (**c**) frequencies in B220$^+$ B cells ad total splenocytes, respectively. Each symbol represents one mouse, and lines denote mean values. Data were pooled from three independent experiments. *P* values by two-tailed unpaired *t* tests. **c** The protocol of CD40L blockade. **d** Spontaneous GCs (top) and SPPCs (bottom) after anti-CD40L blockade or control Ig treatment. Each symbol represents one mouse, and lines denote the mean values. Data were pooled from two independent experiments. *P* values by two-tailed unpaired *t* tests.
