# Supplementary material for: B cell-reactive triad of B cells, follicular helper and regulatory T cells at homeostasis
Source: Cell Res. 2024 Feb 7;34(4):295–308. doi: 10.1038/s41422-024-00929-0 (PMC10978943; doi:10.1038/s41422-024-00929-0)
Supplement: Supplementary file 4 — Supplementary information, Fig. S4 [file 41422_2024_929_MOESM4_ESM.pdf]

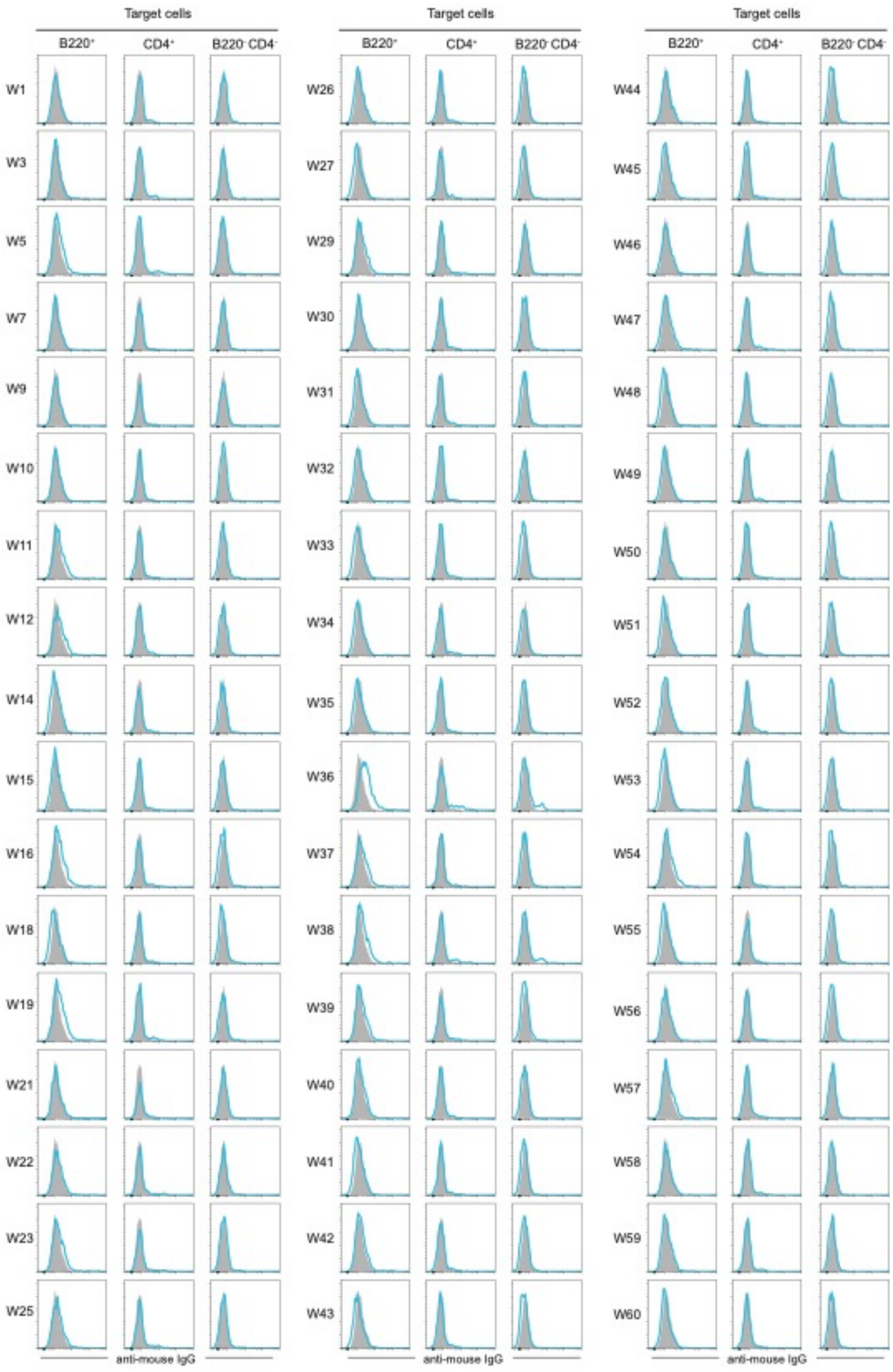
Target cells
B220⁺
CD4⁺
B220⁻CD4⁻
W1
W3
W5
W7
W9
W10
W11
W12
W14
W15
W16
W18
W19
W21
W22
W23
W25
W26
W27
W29
W30
W31
W32
W33
W34
W35
W36
W37
W38
W39
W40
W41
W42
W43
W44
W45
W46
W47
W48
W49
W50
W51
W52
W53
W54
W55
W56
W57
W58
W59
W60
anti-mouse IgG

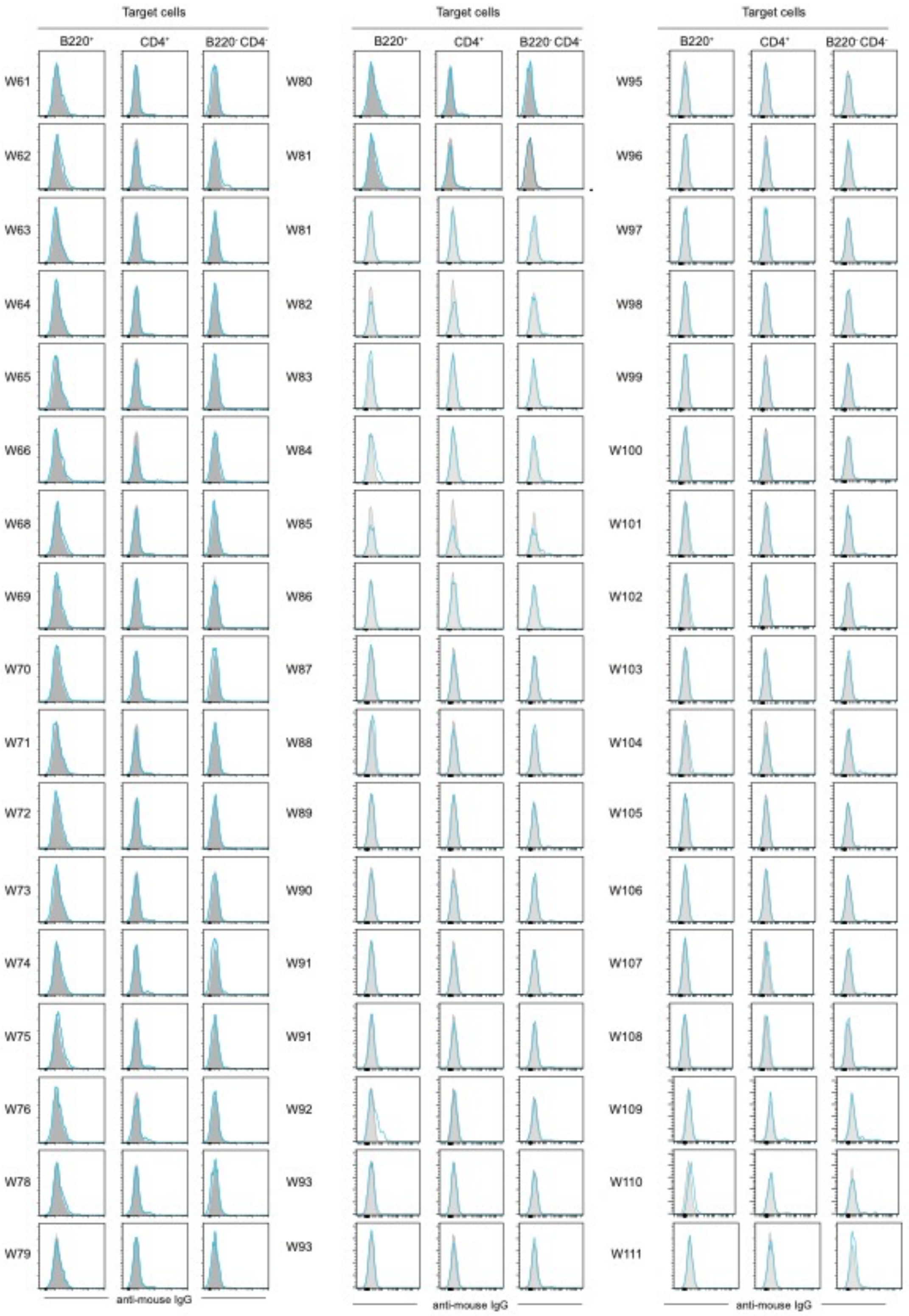
Target cells
B220^{+}
CD4^{+}
B220^{-}CD4^{-}
W61
W62
W63
W64
W65
W66
W68
W69
W70
W71
W72
W73
W74
W75
W76
W78
W79
anti-mouse IgG
Target cells
B220^{+}
CD4^{+}
B220^{-}CD4^{-}
W80
W81
W81
W82
W83
W84
W85
W86
W87
W88
W89
W90
W91
W91
W92
W93
W93
anti-mouse IgG
Target cells
B220^{+}
CD4^{+}
B220^{-}CD4^{-}
W95
W96
W97
W98
W99
W100
W101
W102
W103
W104
W105
W106
W107
W108
W109
W110
W111
anti-mouse IgG

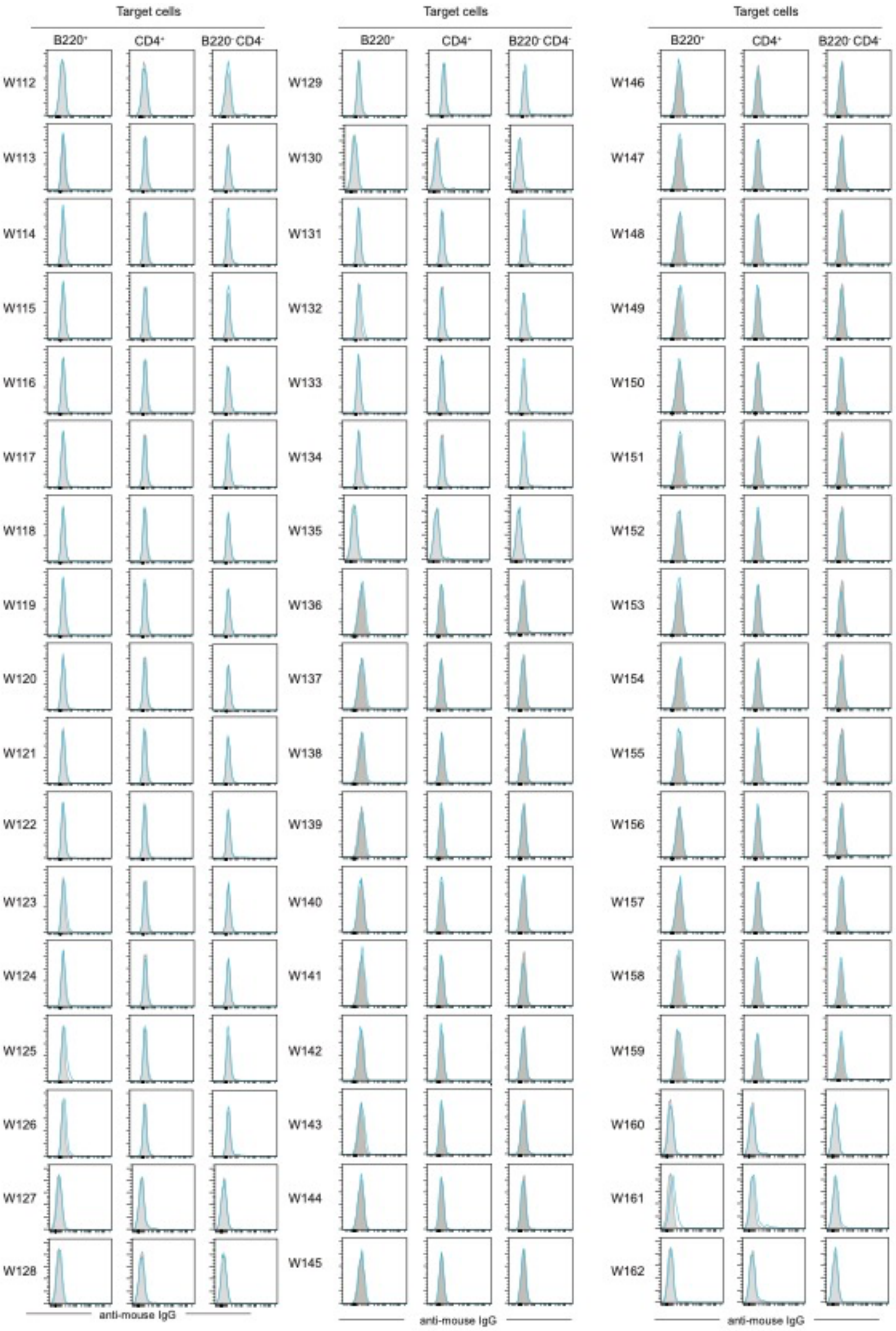
Target cells
B220+
CD4+
B220- CD4-
W112
W113
W114
W115
W116
W117
W118
W119
W120
W121
W122
W123
W124
W125
W126
W127
W128
W129
W130
W131
W132
W133
W134
W135
W136
W137
W138
W139
W140
W141
W142
W143
W144
W145
W146
W147
W148
W149
W150
W151
W152
W153
W154
W155
W156
W157
W158
W159
W160
W161
W162
anti-mouse IgG

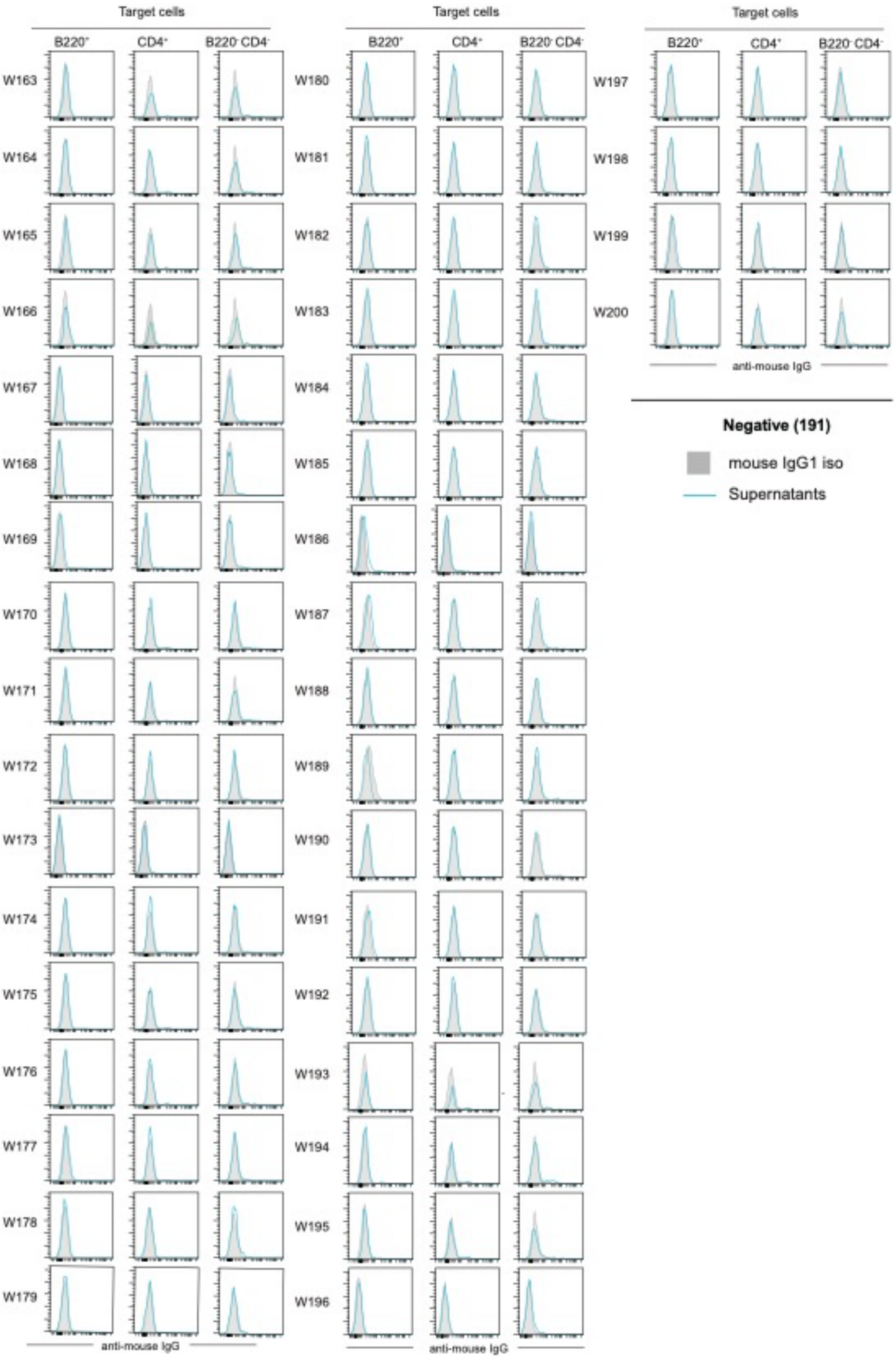
Target cells
B220+
CD4+
B220-CD4-
W163
W164
W165
W166
W167
W168
W169
W170
W171
W172
W173
W174
W175
W176
W177
W178
W179
W180
W181
W182
W183
W184
W185
W186
W187
W188
W189
W190
W191
W192
W193
W194
W195
W196
W197
W198
W199
W200
anti-mouse IgG
Negative (191)
mouse IgG1 iso
Supernatants

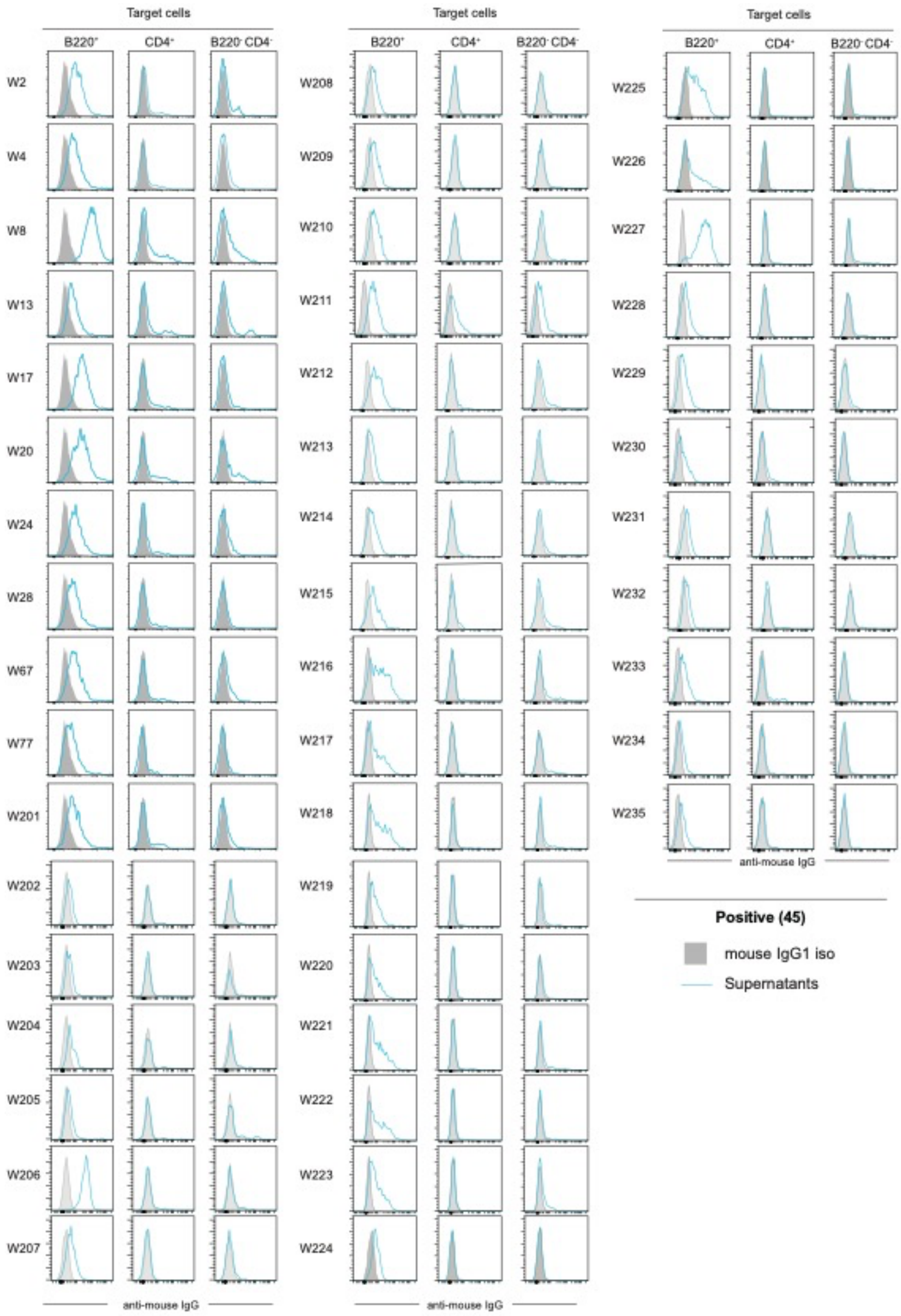
Target cells
B220+
CD4+
B220- CD4-
W2
W4
W8
W13
W17
W20
W24
W28
W67
W77
W201
W202
W203
W204
W205
W206
W207
anti-mouse IgG
Target cells
B220+
CD4+
B220- CD4-
W208
W209
W210
W211
W212
W213
W214
W215
W216
W217
W218
W219
W220
W221
W222
W223
W224
anti-mouse IgG
Target cells
B220+
CD4+
B220- CD4-
W225
W226
W227
W228
W229
W230
W231
W232
W233
W234
W235
anti-mouse IgG
Positive (45)
mouse IgG1 iso
Supernatants

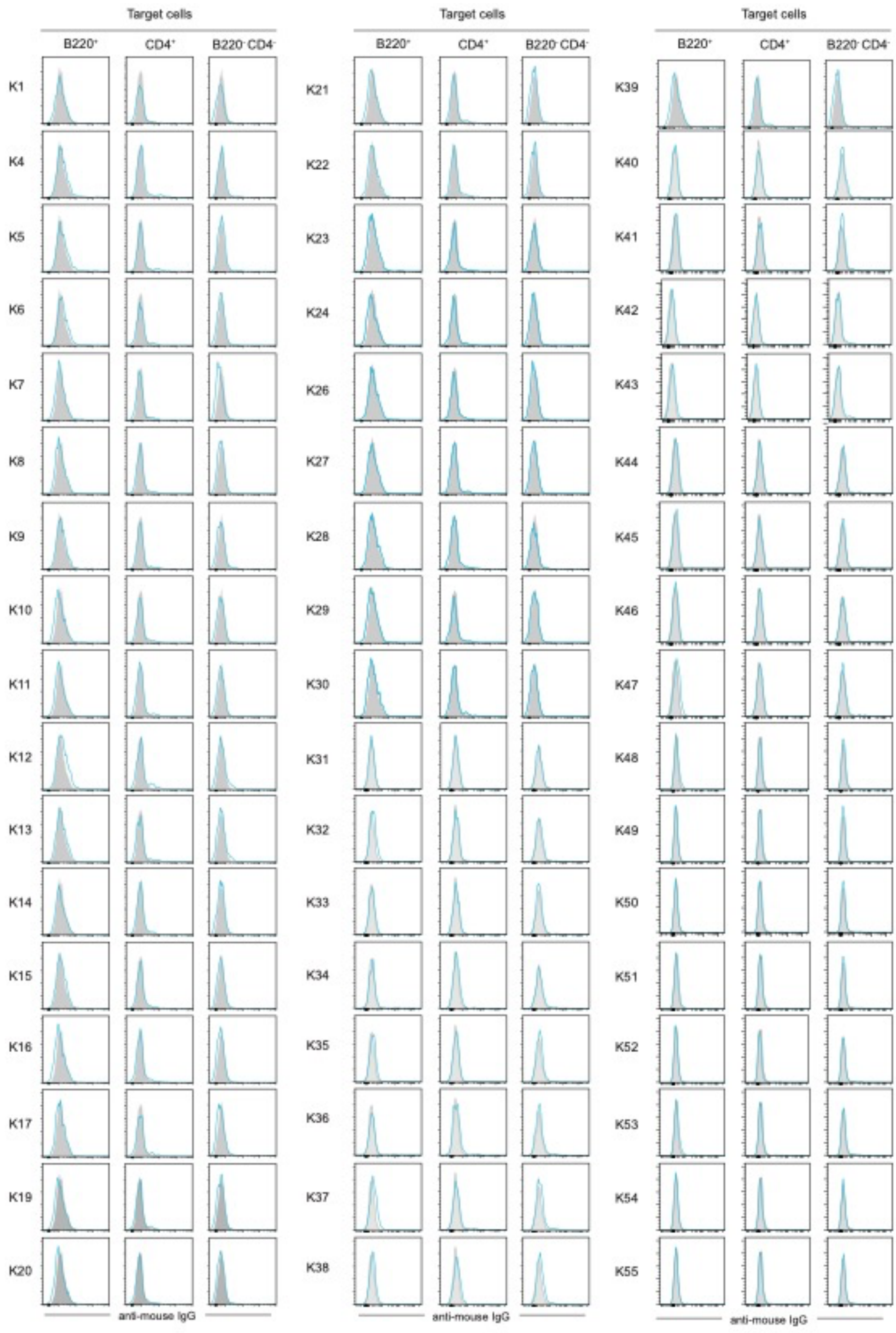
Target cells
B220⁺
CD4⁺
B220⁻CD4⁻
K1
K4
K5
K6
K7
K8
K9
K10
K11
K12
K13
K14
K15
K16
K17
K19
K20
K21
K22
K23
K24
K26
K27
K28
K29
K30
K31
K32
K33
K34
K35
K36
K37
K38
K39
K40
K41
K42
K43
K44
K45
K46
K47
K48
K49
K50
K51
K52
K53
K54
K55
anti-mouse IgG

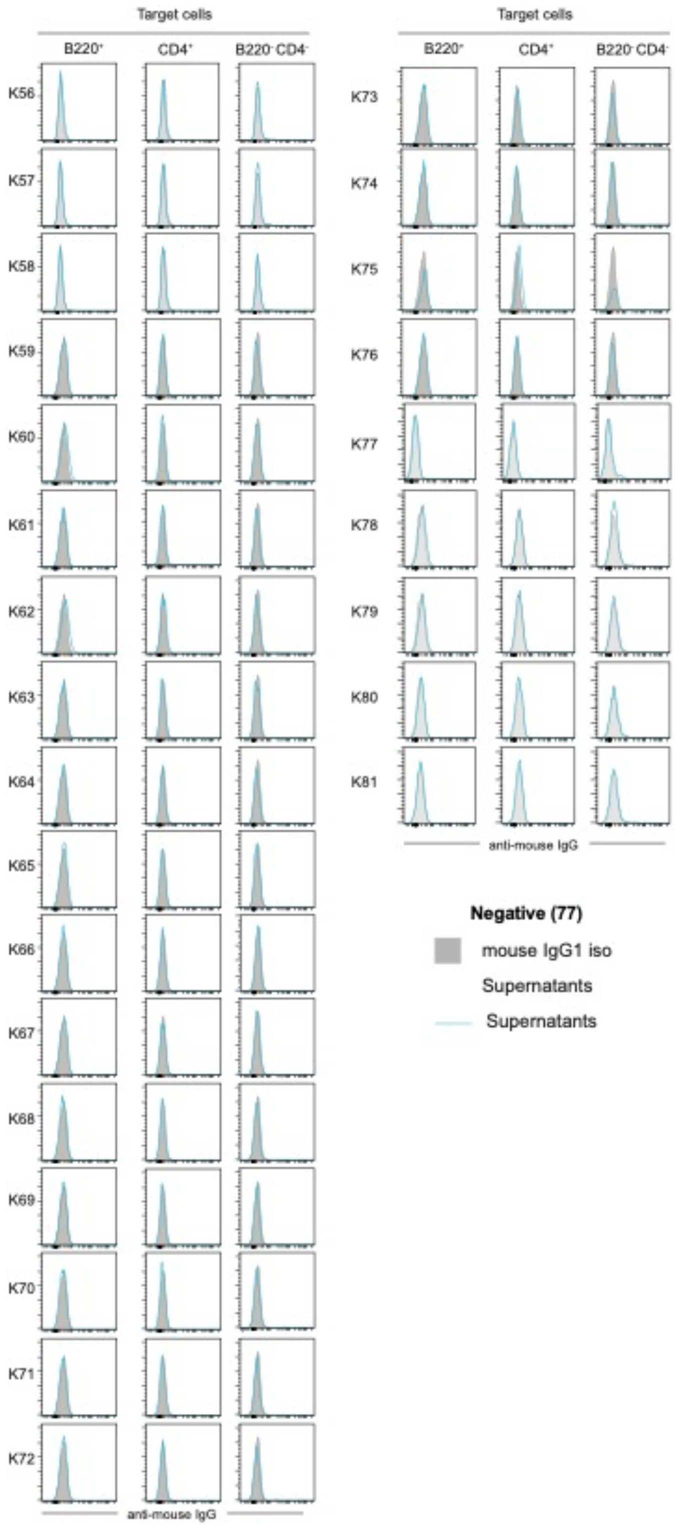
Target cells
B220+
CD4+
B220-CD4-
K56
K57
K58
K59
K60
K61
K62
K63
K64
K65
K66
K67
K68
K69
K70
K71
K72
anti-mouse IgG
Target cells
B220+
CD4+
B220-CD4-
K73
K74
K75
K76
K77
K78
K79
K80
K81
anti-mouse IgG
Negative (77)
mouse IgG1 iso
Supernatants
Supernatants

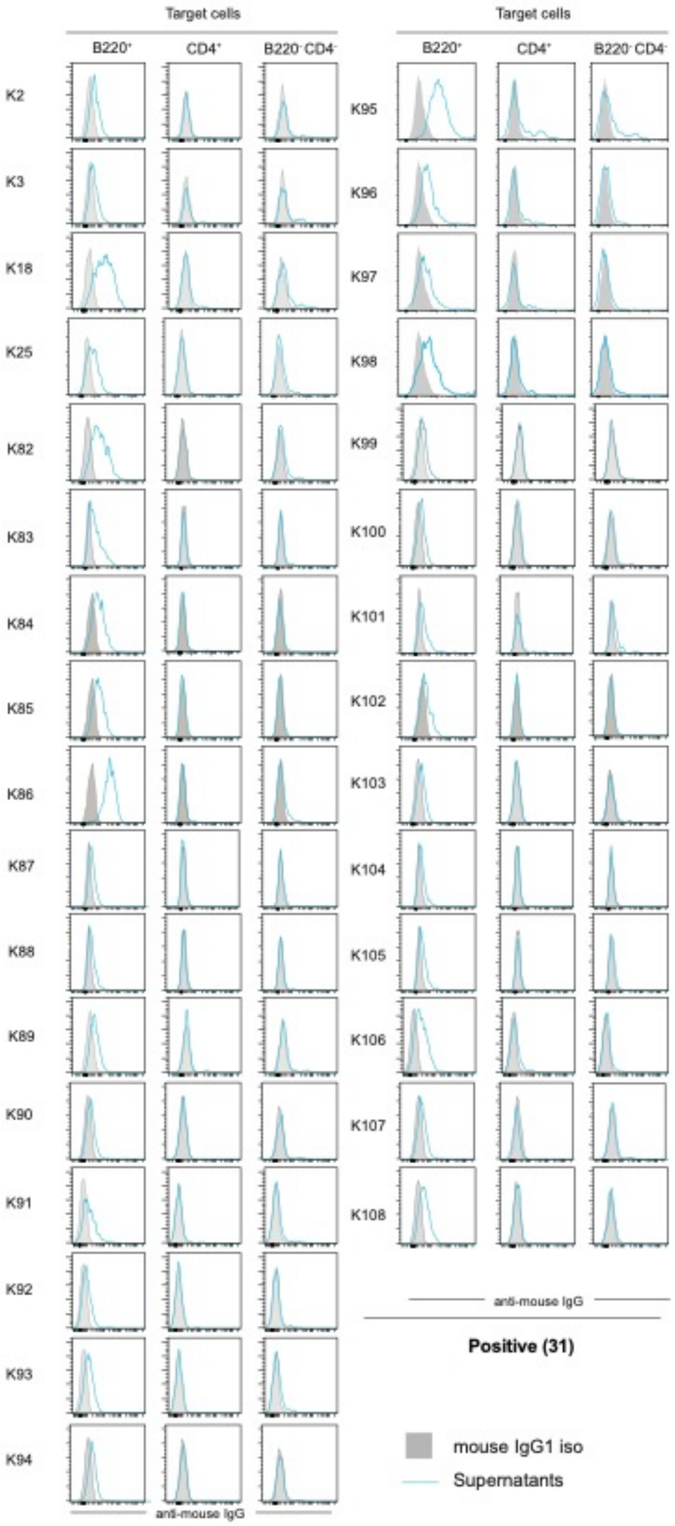

Supplementary information, Fig. S4 Histogram profiles of B or T cells stained with supernatants of single-GC culture.

There were 191 negative (Supplementary information, Fig. S4-1-4) and 45 positive (Supplementary information, Fig. S4-5) clones from T_{FR}-sufficient GCs, 77 negative (Supplementary information, Fig. S4-6-7) and 31 positive (Supplementary information, Fig. S4-8) clones from T_{FR}-insufficient GCs.
