# Supplementary material for: B cell-reactive triad of B cells, follicular helper and regulatory T cells at homeostasis
Source: Cell Res. 2024 Feb 7;34(4):295–308. doi: 10.1038/s41422-024-00929-0 (PMC10978943; doi:10.1038/s41422-024-00929-0)
Supplement: Supplementary file 5 — Supplementary information, Fig. S5 [file 41422_2024_929_MOESM5_ESM.pdf]

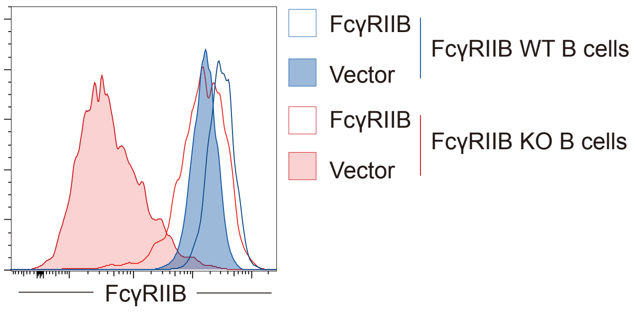

Supplementary information, Fig. S5 FcγRIIB expression on B cells.
Surface FcγRIIB staining of wildtype (WT) or FcγRIIB-deficient (KO) B cells, retrovirally transduced with FcγRIIB isoform 1 or 2.
