# Supplementary material for: B cell-reactive triad of B cells, follicular helper and regulatory T cells at homeostasis
Source: Cell Res. 2024 Feb 7;34(4):295–308. doi: 10.1038/s41422-024-00929-0 (PMC10978943; doi:10.1038/s41422-024-00929-0)
Supplement: Supplementary file 6 — Supplementary information, Fig. S6 [file 41422_2024_929_MOESM6_ESM.pdf]

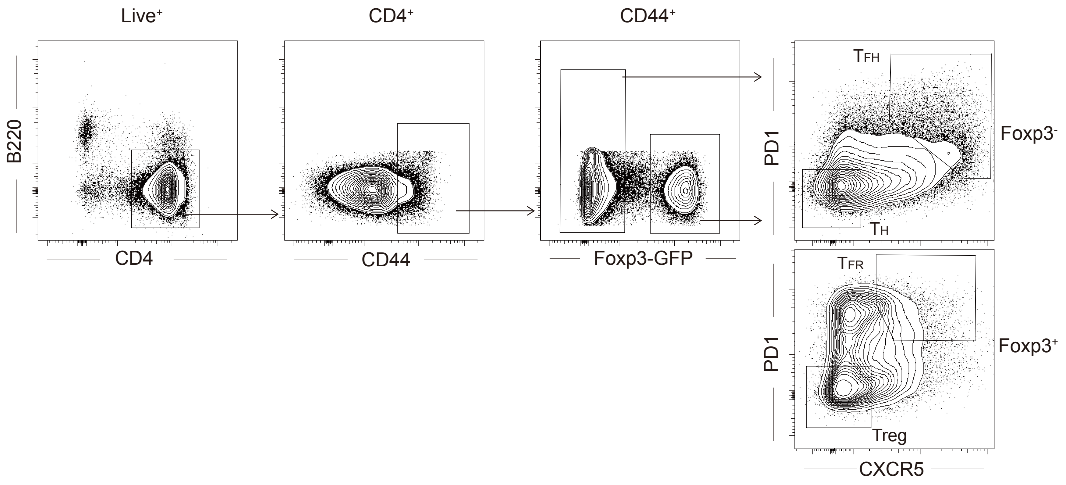

Supplementary information, Fig. S6 Sorting strategy for single-cell TCR sequencing.

The sorting strategy to isolate non-T_{FH} activated cells, T_{FH} cells, conventional Treg cells and T_{FR} cells from Microbead-enriched $CD4^+$ T cells of FoxP3-IRES-GFP mice.
