# Supplementary material for: B cell-reactive triad of B cells, follicular helper and regulatory T cells at homeostasis
Source: Cell Res. 2024 Feb 7;34(4):295–308. doi: 10.1038/s41422-024-00929-0 (PMC10978943; doi:10.1038/s41422-024-00929-0)
Supplement: Supplementary file 7 — Supplementary information, Fig. S7 [file 41422_2024_929_MOESM7_ESM.pdf]

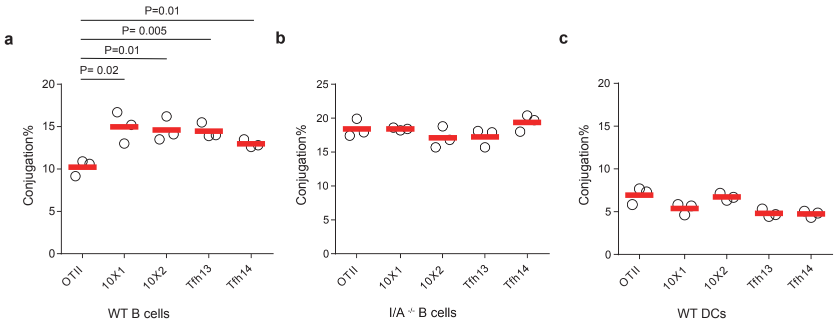

Supplementary information, Fig. S7 Conjugate formation between TCR-transduced T cells and indicated APCs.

Frequencies of polyclonal B6 $CD4^+$ T cells transduced with indicated TCRs conjugating with wildtype B cells (**a**), class II MHC-deficient B cells (**b**) or wildtype DCs (**c**). Each symbol represents one of the triplicated samples. One of two independent experiments with similar results is shown. *P* values by two-tailed unpaired *t* tests.
