# Supplementary material for: B cell-reactive triad of B cells, follicular helper and regulatory T cells at homeostasis
Source: Cell Res. 2024 Feb 7;34(4):295–308. doi: 10.1038/s41422-024-00929-0 (PMC10978943; doi:10.1038/s41422-024-00929-0)
Supplement: Supplementary file 8 — Supplementary information, Fig. S8 [file 41422_2024_929_MOESM8_ESM.pdf]

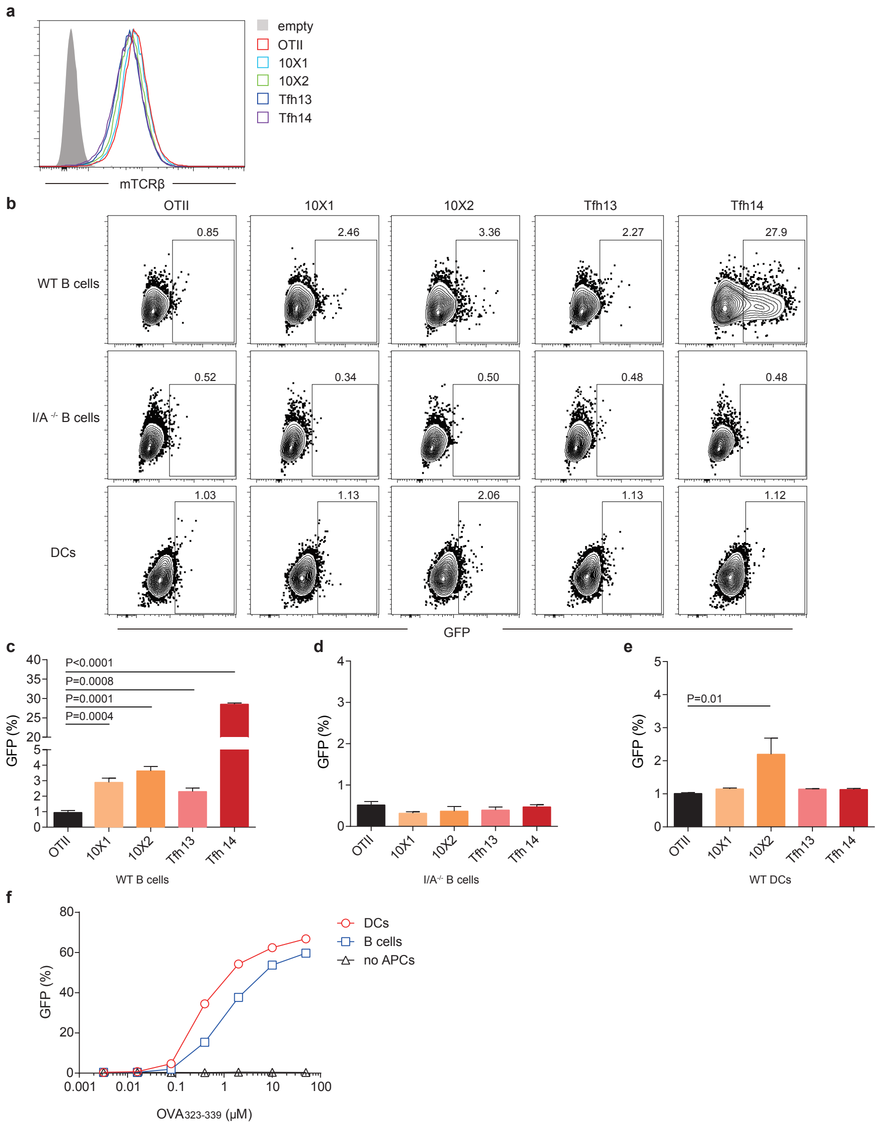

Supplementary information, Fig. S8 Stimulation of TCR-reconstituted hybridoma with indicated APCs.

a Histograms of surface TCRβ expression by mhCD4-NFAT-GFP hybridoma that were transduced with indicated αβ TCRs. **b** Representative contour plots showing GFP^+ cells in hybridoma transduced with indicated TCRs after co-culturing with wildtype B cells, class II MHC-deficient B cells and DCs for 24 h, respectively. **c-e** Summary statistics of GFP^+% hybridoma transduced with indicated TCRs after co-culturing with wildtype B cells (**c**), class II MHC deficient B cells (**d**) or wildtype DCs (**e**). One of two independent experiments with similar results is shown. Bars are STDs of triplicated wells. *P* values by unpaired *t* tests. **f** Summary of GFP^+% of OTII TCR-transduced hybridoma cells after being co-cultured with wildtype B cells or DCs in the presence of the OVA peptide at indicated concentrations.
