# Supplementary material for: B cell-reactive triad of B cells, follicular helper and regulatory T cells at homeostasis
Source: Cell Res. 2024 Feb 7;34(4):295–308. doi: 10.1038/s41422-024-00929-0 (PMC10978943; doi:10.1038/s41422-024-00929-0)
Supplement: Supplementary file 9 — Supplementary information, Fig. S9 [file 41422_2024_929_MOESM9_ESM.pdf]

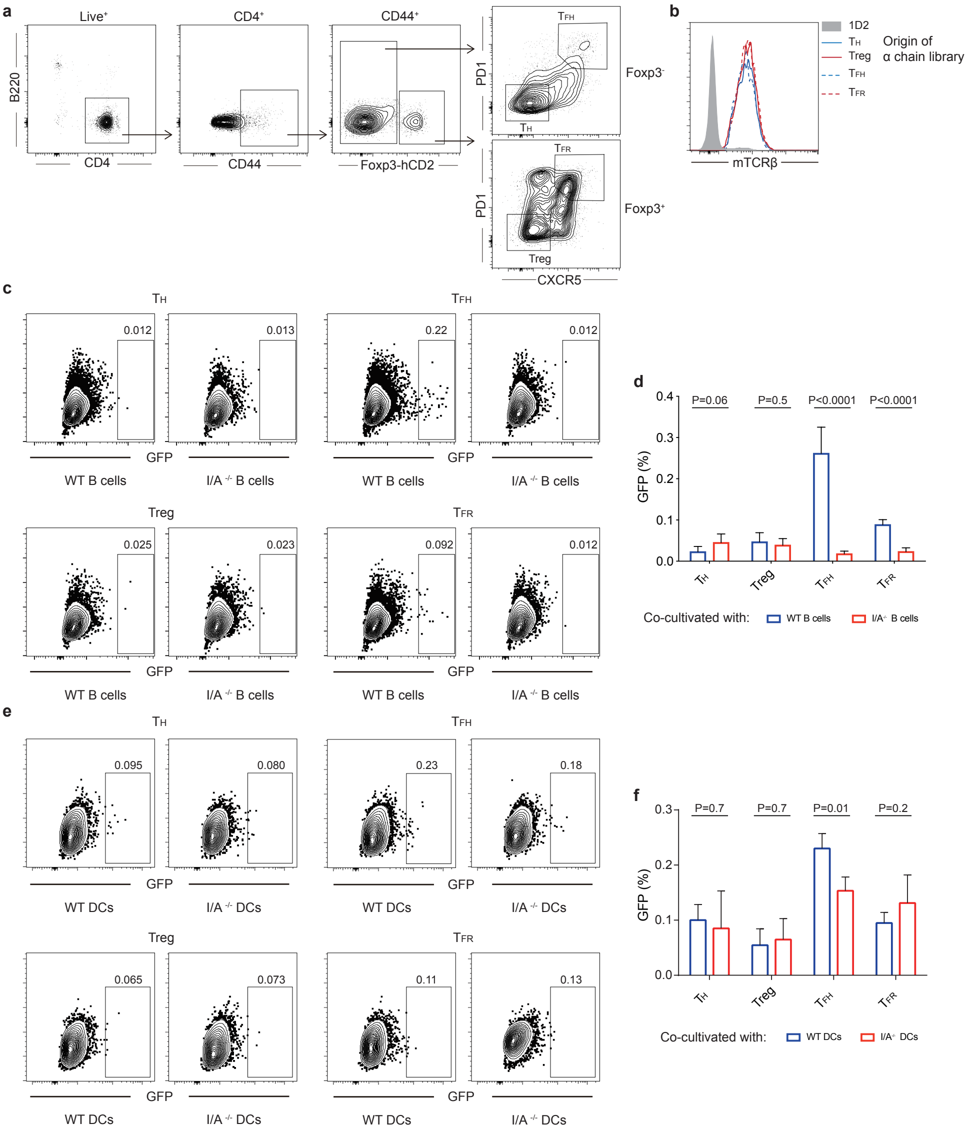

Supplementary information, Fig. S9 Stimulation of hybridoma reconstituted with TCR libraries from the spontaneous condition.

a The strategy of sorting non-T_{FH} (FoxP3-hCD2$^-$CD4$^+$CD44$^+$CXCR5$^-$PD-1$^-$) activated (T_H) cells, T_{FH} (FoxP3- hCD2$^-$CD4$^+$CD44$^+$CXCR5$^+$PD-1$^+$) cells, conventional Treg (FoxP3- hCD2$^+$CD4$^+$CD44$^+$CXCR5$^-$PD-1$^-$) cells and T_{FR} (FoxP3-hCD2$^+$CD4$^+$CD44$^+$CXCR5$^+$PD-1$^+$) cells from Microbead-enriched splenic CD4$^+$ T cells of *Foxp3*$^{hCD2/y}$*Tcrb*$^{1D2/1D2}$ or *Foxp3*$^{hCD2/hCD2}$*Tcrb*$^{1D2/1D2}$ mice. **b** Histograms of surface TCRβ expression by 1D2β-mhCD4-NFAT-GFP hybridoma that were transduced with TCRα libraries isolated from indicated T cell populations. **c-d** Representative contour plots showing GFP$^+$ cells (**c**) and summary statistics of GFP$^+$% (**d**) in hybridoma transduced with indicated TCR libraries after co-culturing with wildtype (blue) or class II MHC-deficient (red) B cells for 24 h. **e-f** Representative contour plots showing GFP$^+$ cells (**e**) and summary statistics of GFP$^+$% (**f**) in hybridoma transduced with indicated TCR libraries after co-culturing with wildtype (blue) or class II MHC-deficient (red) DCs for 24 h. One of two independent experiments with similar results is shown. Bars are STDs of quadruplicated wells. *P* values by unpaired *t* tests.
