# Supplementary material for: B cell-reactive triad of B cells, follicular helper and regulatory T cells at homeostasis
Source: Cell Res. 2024 Feb 7;34(4):295–308. doi: 10.1038/s41422-024-00929-0 (PMC10978943; doi:10.1038/s41422-024-00929-0)
Supplement: Supplementary file 10 — Supplementary information, Fig. S10 [file 41422_2024_929_MOESM10_ESM.pdf]

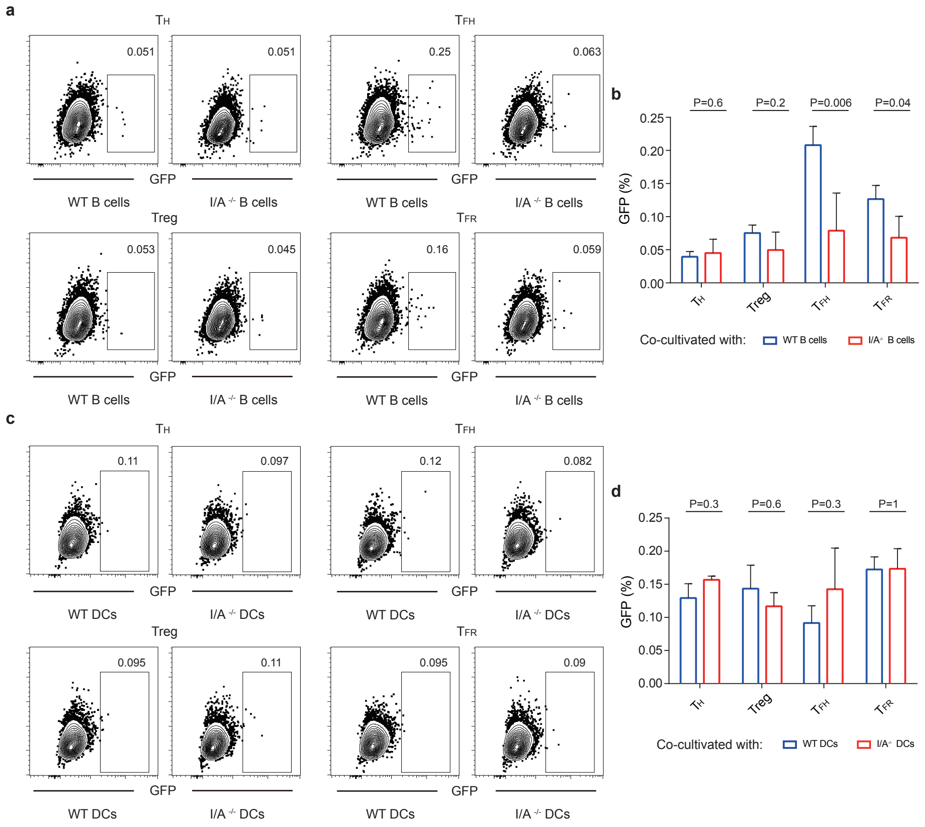

Supplementary information, Fig. S10 Stimulation of hybridoma reconstituted with TCR libraries from the immunized condition.

a-b Representative contour plots showing GFP$^+$ cells (**a**) and summary statistics of GFP$^+$% (**b**) in hybridoma transduced with indicated TCR libraries after co-culturing with wildtype (blue) or class II MHC-deficient (red) B cells for 24 h. **c-d** Representative contour plots showing GFP$^+$ cells (**c**) and summary statistics of GFP$^+$% (**d**) in hybridoma transduced with indicated TCR libraries after co-culturing with wildtype (blue) or class II MHC-deficient (red) DCs for 24 h. One of two independent experiments with similar results is shown. Bars are STDs of quadruplicated wells. *P* values by unpaired *t* tests.
