# Supplementary material for: B cell-reactive triad of B cells, follicular helper and regulatory T cells at homeostasis
Source: Cell Res. 2024 Feb 7;34(4):295–308. doi: 10.1038/s41422-024-00929-0 (PMC10978943; doi:10.1038/s41422-024-00929-0)
Supplement: Supplementary file 11 — Supplementary information, Table S1 [file 41422_2024_929_MOESM11_ESM.pdf]

Supplementary information, Table S1. Spontaneous GC-derived recombinant antibodies that react to B cell surface molecules.

	IgL				IgH				
Clone name	V	J	CDR3 aa	mutation	V	D	J	CDR3 aa	mutation
UNW10	IGKV2-109*01	IGKJ1*01	AQNLELPWT	0	IGHV1-55*01	IGHD1-1*01	IGHJ1*03	ARKGIYYYGSSHWYFDV	0
UNKO8	IGKV9-124*01	IGKJ1*01	LQYASYPRT	0	IGHV1-59*01	N/A	IGHJ2*01	ARWGFFDY	0
K3H12	IGKV4-50*01	IGKJ2*01	QQFTSSPYT	CDR3:2	IGHV1-9*01	N/A	IGHJ2*01	AREGRSYYFDY	0
W2H5	IGKV1-117*01	IGKJ2*01	FQGSHVPYT	FR1:1	IGHV1-26*01	IGHD2-1*01, IGHD2-10*01, IGHD2-10*02	IGHJ3*01	ARSPYGNYVYVAWFAY	0
W3H5	IGKV4-72*01	IGKJ2*01	QQWSSNPPT	0	IGHV1-42*01	IGHD3-2*02	IGHJ3*01	ARRTAQTFAY	0
K3H5	IGLV2*02	IGLJ2*01	ALWYSTHYV	0	IGHV1-64*01	IGHD2-5*01, IGHD2-6*01	IGHJ3*01	SYYSNYVGFAY	0
K3F10	IGLV1*01	IGLJ1*01	ALWYSNHLV	0	IGHV1-66*01	N/A	IGHJ3*01	ASETLAY	0
W3C2	IGLV1*01	IGLJ1*01	ALWYSNHLV	0	IGHV9-3*01	IGHD1-1*01	IGHJ2*01	ARLYYGSSYVGGN	CDR3:1
W3A11	IGKV6-15*01	IGKJ4*01	QQYNSYPFT	0	IGHV1-53*01	IGHD2-1*01, IGHD2-13*01, IGHD2-2*01	IGHJ2*01	ARRIYPYYFDY	0
W3A7	IGKV8-28*01	IGKJ2*01	QNDHSYPYT	0	IGHV5-6*02	IGHD1-1*01	IGHJ4*01	ARRYGSSHYYAMDY	0
W2H1	IGKV19-93*01	IGKJ5*01	LQYDNLLLT	0	IGHV9-3*01	IGHD1-1*01	IGHJ3*01	ARRVYYGSSRSPFAY	0
W2A3	IGKV5-37*01	IGKJ1*01	LQGYSTPWT	0	IGHV2-5*01	IGHD1-1*01	IGHJ4*01	AKFPYGSSYAMDY	0
K2A1	IGKV12-98*01	IGKJ4*01	QQLYSTPFT	0	IGHV1-26*01	IGHD2-3*01	IGHJ2*01	ARDDGWV	0
