# Supplementary material for: B cell-reactive triad of B cells, follicular helper and regulatory T cells at homeostasis
Source: Cell Res. 2024 Feb 7;34(4):295–308. doi: 10.1038/s41422-024-00929-0 (PMC10978943; doi:10.1038/s41422-024-00929-0)
Supplement: Supplementary file 12 — Supplementary information, Table S2 [file 41422_2024_929_MOESM12_ESM.pdf]

**Supplementary information, Table S2. Four TCRs from spontaneous $T_{FH}$ and $T_{FR}$ cells.**

| Clone name | Exp.Mouse\Cell[a] | TCRα | | | TCRβ | | | |
|---|---|---|---|---|---|---|---|---|
| | | V | J | CDR3 | V | D | J | CDR3 |
| 10X1 | meta-3\ Tfh<br>meta-1\ Tfr | TRAV5-4 | TRAJ31 | TGTGCTGCAAGTAATAGCAATAAC AGAATCTTCTTT | TRBV13-2 | N/A | TRBJ1-6 | TGTGCCAGCGGTGATTCCTATAATT CGCCCCTCTACTTT |
| 10X2 | 5-4 \ Tfh | TRAV5-4 | TRAJ31 | TGTGCTGCAAGCAATAGCAATAAC AGAATCTTCTTT | TRBV13-2 | N/A | TRBJ1-6 | TGTGCCAGCGGTGATGCATATAATT CGCCCCTCTACTTT |
| | meta-1\ Tfr | TRAV5N-4 | TRAJ31 | TGTGCTGCAAGTAATAGCAATAAC AGAATCTTCTTT | TRBV13-2 | N/A | TRBJ1-6 | TGTGCCAGCGGTGATGCGTATAATT CGCCCCTCTACTTT |
| | 5-1 \ Tfr | TRAV5-4 | TRAJ31 | TGTGCTGCCTCGAATAGCAATAACA GAATCTTCTTT | TRBV13-2 | N/A | TRBJ1-6 | TGTGCCAGCGGTGATGCGTATAATT CGCCCCTCTACTTT |
| | 5-2 \ Treg | TRAV5-4 | TRAJ31 | TGTGCTGCAAGTAATAGCAATAAC AGAATCTTCTTT | TRBV13-2 | N/A | TRBJ1-6 | TGTGCCAGCGGTGATGCTTATAATT CGCCCCTCTACTTT |
| | 5-4 \ Treg | TRAV5-4 | TRAJ31 | TGTGCTGCAAGTAATAGCAATAAC AGAATCTTCTTT | TRBV13-2 | N/A | TRBJ1-6 | TGTGCCAGCGGTGATGCCTATAATT CGCCCCTCTACTTT |
| Tfh13 | meta-3 \ Tfh | TRAV6-7-DV9 | TRAJ42 | TGTGCTCTGAGTGGGGGAGGAAGC AATGCAAAGCTAACCTTC | TRBV15 | TRBD2 | TRBJ2-7 | TGTGCCAGCAGTTTGGGGGGGAGCT CCTATGAACAGTACTTC |
| | 5-1 \ Tfh | TRAV6-7-DV9 | TRAJ42 | TGTGCTCTGAGTGGAGGAGGAAGC AATGCAAAGCTAACCTTC | TRBV15 | TRBD2 | TRBJ2-7 | TGTGCCAGCAGTTTAGGGGGGAGCT CCTATGAACAGTACTTC |
| Tfh14 | 4-3 \ Tfh | TRAV7-4 | TRAJ17 | TGTGCAGCTAGTGCGTCGACTAACA GTGCAGGGAACAAGCTAACTTTT | TRBV12-2 | N/A | TRBJ2-3 | TGTGCCAGCTCTCTCGACTCTAGTG CAGAAACGCTGTATTTT |

[a] Three of 5 sequencing experiments involved cells pooled from 8 mice per batch, and these are termed meta-mouse 1, 2, and 3 (meta-1, 2, 3). In the other two ($4^{th}$ and $5^{th}$) experiments, each of the 8 donor mice was individually barcoded (termed 4-1~8, 5-1~8).
